# Histone demethylase KDM4A promotes endometrial cancer progression through an ERRγ-associated cell-cycle regulatory axis

**DOI:** 10.7150/ijbs.133986

**Published:** 2026-09-03

**Authors:** Junfeng Chen, Xiaoli Wen, Donghai Zhang, Mengyue Zhu, Hong Zhou, Yiran Li, Xiaoping Wan

**Affiliations:** 1 Shanghai Key Laboratory of Maternal Fetal Medicine, Shanghai Institute of Maternal-Fetal Medicine and Gynecologic Oncology, Shanghai First Maternity and Infant Hospital, School of Medicine, Tongji University, Shanghai, 200092, China.; 2 Center for Reproductive Medicine, Shanghai First Maternity and Infant Hospital, Tongji University School of Medicine, Tongji University, Shanghai 200092, China.; 3 Department of Gynecology, Shanghai First Maternity and Infant Hospital, School of Medicine, Tongji University, Shanghai, China.

**Keywords:** endometrial cancer, KDM4A, ERRγ, cell cycle, histone modification

## Abstract

Endometrial cancer (EC) is driven by complex genetic and epigenetic alterations, but the specific chromatin-dependent mechanisms that sustain malignant proliferation remain incompletely understood. In this study, we found that lysine demethylase 4A (KDM4A) was upregulated in EC and promoted cell proliferation, migration, and invasion, accompanied by increased estrogen-related receptor gamma (ERRγ) expression. Mechanistically, KDM4A promoted ERRγ expression in association with reduced enrichment of the repressive H3K9me3 modification at the ESRRG promoter, suggesting that KDM4A may facilitate ESRRG transcription by attenuating H3K9me3-associated repression. Functionally, ERRγ acted as a downstream mediator associated with increased CDK1 transcription and elevated CDK1 and Cyclin B1 protein expression. These findings suggest that the KDM4A-ERRγ axis contributes to G2/M-phase regulation and EC cell proliferation. Importantly, treatment with the KDM4-family inhibitor QC6352 significantly suppressed EC cell growth *in vitro* and reduced xenograft tumor growth *in vivo*. These findings support a model in which KDM4A-associated reduction of H3K9me3 enrichment at the ESRRG promoter contributes to ERRγ upregulation and altered cell-cycle regulation in EC cells. Furthermore, these findings provide preliminary preclinical support for further investigation of KDM4-family inhibition as a potential therapeutic approach in EC.

## Introduction

Endometrial cancer (EC) is a leading gynecological malignancy worldwide, ranking sixth among the most common cancers in women 1, 2. In 2020, the global count of new cases exceeded 410,000, with 97,000 deaths reported 2. EC is expected to see a continued rise in mortality rates over the next few years, distinguishing it as one of the few cancers with an increasing death rate 3. The majority of cases are found in women between the ages of 65 and 75, with obesity significantly influencing both the occurrence and death rates 1, 4. Standard EC treatment involves surgery, radiation therapy, and chemotherapy 5. However, about 15%-20% of patients see their disease return or progress after the first round of treatment 5, 6. Despite the significant advancements in understanding the biological diversity of EC thanks to The Cancer Genome Atlas (TCGA), the main challenge remains its complex pathogenesis, which involves various genetic and epigenetic factors 5-7.

Among numerous factors, histone modification as a crucial epigenetic mechanism plays a significant role in regulating gene expression 8, 9. In recent years, an increasing number of studies have revealed the importance of histone modification in the occurrence and development of EC 10. Epigenetic alterations refer to reversible but heritable modifications in histones and DNA, and are an important component of tumor progression 11, 12. Histone modification regulates gene expression by altering chromatin structure and affecting the accessibility of transcription factors to gene promoters 11-14. Histone demethylation plays a pivotal role in the occurrence and progression of EC 8, 13. Aberrant histone demethylation can lead to the activation of oncogenes or the silencing of tumor suppressor genes, thereby driving tumor progression 12, 13. By delving into the molecular mechanisms of histone modification, we can gain a deeper understanding of the pathological mechanisms underlying EC and offer novel insights and strategies for its treatment 13, 15, 16. The research and development of new drugs targeting this mechanism primarily focus on developing histone demethylase inhibitors, providing a more comprehensive theoretical basis for the precision treatment of EC 15, 16.

The cell cycle is a process of ordered growth, DNA replication, and division of cells, and its core mechanism is precisely controlled by cyclin-dependent kinases and their regulatory factors 17-20. Multiple studies have reported the phenomenon of cell cycle deregulation in various cancers 18, 21. Driven by certain carcinogenic signals, cell cycle-related proteins undergo overexpression and activity imbalance, thereby forcibly driving abnormal cell division, inducing genomic instability, and aiding cells in resisting death, ultimately promoting the malignant transformation of normal cells and tumor progression 22, 23. Dysregulation of the cell cycle is one of the core mechanisms underlying the occurrence and progression of EC 24, 25. The regulation of cell cycle is a frontier area in the research of EC treatment 24-26. Developing small molecule inhibitors targeting key cell cycle proteins and advancing cell cycle-targeted therapeutics represent pivotal future strategies in EC treatment 24, 27, 28.

In this study, we investigated the biological and epigenetic functions of KDM4A in EC. KDM4A expression was associated with reduced H3K9me3 enrichment at the ESRRG promoter and increased ERRγ expression, suggesting that KDM4A may facilitate ESRRG transcription by attenuating H3K9me3-associated repression. ERRγ was further associated with increased CDK1 transcription, elevated Cyclin B1 and CDK1 expression, and altered G2/M-phase distribution. In addition, QC6352 exerted antitumor effects in EC cell and subcutaneous xenograft models, supporting further investigation of KDM4-family demethylase inhibition in EC.

## Materials and Methods

### Data source

The Cancer Genome Atlas (TCGA) database (https://cancergenome.nih.gov) was used to download transcriptome and clinical data for UCEC patients. Protein expression profiles for normal tissues and UCEC were obtained from the UALCAN database (https://ualcan.path.uab.edu/). The gene expression profiles were downloaded from Gene Expression Omnibus (GEO) database (https://www.ncbi.nlm.nih.gov/gds). The Kaplan-Meier Plotter dataset (http://kmplot.com/analysis/) was used to analyze the correlation between gene expression and survival time of EC patients.

### Tissue samples

The research gathered human EC samples along with their corresponding normal tissues from Shanghai First Maternity and Infant Hospital. All procedures were carried out following the guidelines set by the Ethics Committee of Shanghai First Maternity and Infant Hospital (Approval Number: KS23284). Informed consent was obtained from all patients prior to sample collection.

### Cell culture and reagents

The HEC1-B cell line (Cat: FH0306), Ishikawa cell lines (Cat: FH0305) and the HEK293T cell line (Cat: FH0244) were purchased from Shanghai Fuheng Biotechnology and authenticated by STR profiling. The cells were maintained in culture under standard-conditions using DMEM/F12 medium (absin, abs9560) supplemented with 10% fetal bovine serum (NEST, Cat:209111) and 1% penicillin-streptomycin (absin, abs9244). All cells were grown in an incubator maintained at 37°C with a CO2 concentration of 5%. The KDM4 inhibitor, QC6352 (HY-104048), was purchased from MCE (New Jersey, USA).

### RNA isolation and quantitative reverse transcription-polymerase chain reaction (RT-qPCR)

Total RNA was extracted from cultured cells or frozen endometrial tumor tissues using Tissue/Cell RNA Rapid Extraction Kit (absin, abs60027). Afterward, RNA was converted into cDNA using the ABScript III RT Master Mix for qPCR with gDNA Remover (ABclonal, RK20429). qPCR detection was performed using 2X Universal SYBR Green Fast qPCR Mix (ABclonal, RK21203) on a StepOnePlus real-time quantitative PCR system, strictly following the standard protocol provided by the manufacturer. Each sample underwent three replicate measurements and was normalized based on the expression level of the reference GAPDH gene. Relative gene expression was calculated using the 2^-ΔΔCt^ method, with GAPDH serving as the internal reference.

### Western blotting

Lyse cell or tissue samples on ice using RIPA lysis buffer (absin, abs9229) containing general protease inhibitors (absin, abs9161), centrifuge and collect the supernatant. The protein concentration was determined using BCA protein quantification kit (absin, abs9232) and adjusted to a uniform concentration with 5× SDS-PAGE loading buffer (absin, abs9829), followed by denaturation in boiling water bath. Subsequently, SDS-PAGE electrophoresis was performed by adding equal amounts of protein samples and multicolor protein markers to precast gels (absin, abs9604), and electrophoresis was performed in Tris-Glycine-SDS electrophoresis buffer (absin, abs951) under the conditions of stacking gel 80 V and resolving gel 120 V. After electrophoresis, the protein was transferred from the gel to the PVDF membrane (absin, abs932) activated with methanol in advance by wet transfer method, and then transferred to the ice bath at a constant current of 300 mA for 60-90 min. After the membrane transfer is completed, block with TBST solution (absin, abs952) containing 5% skim milk (absin, abs9175) at room temperature for 1 hour, and then incubate overnight with specific primary antibody at 4℃. After washing with TBST, incubate with the corresponding HRP labeled secondary antibody at room temperature for 1 hour. Finally, chemiluminescent ECL detection reagent (abclonal, RM02867) was used for development, and images were collected using a chemiluminescence imaging system. The ImageJ software was used to perform semiquantitative analysis based on the band gray value ratio of the target protein to internal reference protein. The antibodies used in this study are listed in Supplementary Table S1.

### Immunohistochemistry (IHC) staining

Formalin-fixed, paraffin-embedded (FFPE) specimens from human EC tissues and matched adjacent non-tumor endometrium, as well as xenograft tumors harvested from a mouse subcutaneous implantation model, were sectioned at 4 μm thickness. Slides were deparaffinized in xylene and rehydrated through graded ethanol. Endogenous peroxidase activity was blocked with 3% hydrogen peroxide (absin, abs9333) for 10 min at room temperature, followed by antigen retrieval in citrate antigen retrieval solution (absin, abs9248) using microwave heating for 20 min. After cooling to room temperature, sections were rinsed with PBS and blocked with 10% Goat serum (absin, abs933) for 60 min. The sections were then incubated overnight at 4°C with primary antibodies. After washing, sections were incubated with HRP-conjugated secondary antibodies for 60 min at room temperature. After immunoreactivity was visualized with DAB chromogen and counterstained with hematoxylin, the slides were dehydrated, cleared, and mounted. IHC staining was independently assessed using a semiquantitative scoring system. Briefly, staining intensity was scored on a four-tier scale: 0 for negative staining, 1 for weak staining, 2 for moderate staining, and 3 for strong staining. The percentage of positive tumor cells was recorded for each sample. The final IHC score was calculated by multiplying the staining-intensity score by the percentage of positively stained cells.

### Multiple fluorescence immunohistochemical staining (mIHC)

Cell culture samples were washed with PBS, fixed in 4% paraformaldehyde for 15 minutes, and then washed three times with PBS. After permeabilization with 0.3% Triton X-100 (absin, abs9149) for 10-15 minutes, the samples were blocked with 5% fetal bovine serum (FBS) for 1 hour. The samples were then incubated with the primary antibody overnight at 4°C, followed by three PBS washes. Next, the samples were incubated with the fluorescently labeled secondary antibody for 1 hour at room temperature, with the use of fluorescent dye and concentration according to the manufacturer's instructions (absin, abs50012), and washed three times with PBS. To avoid signal overlap, each fluorescent dye used distinct excitation and emission wavelengths. The nuclei were labeled with DAPI in an anti-fade solution. Samples were observed and captured under confocal microscope.

### Plasmid and lentivirus infection

All transfections were performed using the HighGene Transfection Reagent (ABclonal, RM09014) according to the manufacturer's instructions. The plasmids for KDM4A and ESRRG were designed by Generay Biotechnology Co., Ltd. (Shanghai, China), with scrambled sequences used as the negative control. Packaging was carried out using the three-plasmid system psPAX2 and pMD2G. The lentivirus supernatant was collected 48 and 72 hours post-transfection, filtered, and concentrated using the Lenti-X concentrator (Takara, Japan) according to the manufacturer's protocol. Concentrated lentivirus was used to transduce HEC1-B and Ishikawa cells, and stable cell lines were obtained after selection with 2 μg/mL puromycin for two weeks. The primers employed in this research are detailed in Supplementary Table S2.

### Proliferation assay

CCK-8 assay: Seed the cells required for the experiment into 96-well plates (1500 cells per well) and incubate them for 0, 24, 48, 72, and 96 hours, respectively. Add CCK-8 reagent (absin, abs50003) to each well according to the manufacturer's instructions and incubate the plates in a 5% carbon dioxide incubator at 37°C for 2 hours. Measure the absorbance at a wavelength of 450 nm using a microplate reader.

Colony Forming Assay: Seed 1000 cells into a 6-well plate, incubate at 37°C for 2 weeks, fix with 4% paraformaldehyde (absin, abs9179), and then stain with 0.5% crystal violet. Count colonies containing at least 50 cells.

EdU assay: According to the instructions (Epizyme Biotech, CX003), add EdU working solution with a final concentration of 10 μM to the cell culture medium and incubate for 2 hours for labeling. Then, discard the culture medium, wash the cells with PBS, fix them with 4% paraformaldehyde, and treat them with permeabilization solution. Next, use a light-protected Click Additive Solution to allow the fluorescent group to bind to EdU. After the reaction is complete, thoroughly wash the cells, then counterstain the nuclei, and finally photograph and analyze the proportion of EdU-positive cells using a fluorescence microscope.

### Transwell assay

Migration assay: In the upper chamber, 50,000 cells will be seeded in 200 µL of serum-free medium in a Transwell chamber (Cat. #3422, Corning, USA). Invasion assay: After pre-coating and solidifying a 1:9 diluted Matrigel (absin, abs9491), 200,000 cells were seeded into the upper chamber of a 24-well Transwell chamber, using 200 µL of serum-free medium. Add 800 µL of complete medium to the lower chamber, and then incubate the culture plate in a 37°C humidified incubator with 5% CO2. Fix the cells on the lower surface of the membrane with 1% formaldehyde, stain with 1% crystal violet, remove the non-migrated cells on the surface of the upper chamber with a cotton swab, and count under a microscope.

### Cell cycle assay

For cell cycle analysis, we conducted experiments using a cell cycle detection kit (absin, abs50005). The cells required for the experiment were resuspended in 200 µL of PBS buffer and filtered through a cell screen to obtain a single-cell suspension. The suspension was fixed overnight at 4°C with precooled 70% ethanol. The cells were then washed three times with PBS, followed by treatment with RNaseA and propidium iodide (PI) at 37°C in the dark for 30 minutes. Finally, analysis was performed by flow cytometry to monitor cell cycle distribution.

### Chromatin Immunoprecipitation (ChIP), ChIP-seq and ChIP-qPCR assays

Chromatin immunoprecipitation (ChIP) assays were performed using a ChIP Kit (absin, abs50034) according to the manufacturer's instructions. Briefly, cells were cross-linked with 1% formaldehyde for 5 min at room temperature and quenched with 125 mM glycine. Nuclei were isolated, and chromatin was fragmented by enzymatic digestion. An aliquot of fragmented chromatin was reserved as input DNA, while the remaining chromatin was incubated overnight at 4°C with a ChIP-grade anti-H3K9me3 antibody or normal IgG as a negative control. Protein G magnetic beads were subsequently added and incubated for 2 h to capture immune complexes. After washing and elution, immunoprecipitated DNA was purified for downstream analyses. For ChIP-seq analysis, purified DNA was used for library preparation with the NEBNext Ultra II DNA Library Prep Kit (New England Biolabs, E7103). Libraries were sequenced on the Illumina NovaSeq 6000 platform to generate paired-end 150-bp reads. Raw reads were quality-checked using FastQC and aligned to the human reference genome (hg38) with Bowtie2. Peak calling was performed using MACS2 using matched input DNA as controls. Differential enrichment analysis between the OE and NC groups was conducted using DiffBind, and enrichment tracks were visualized using Integrative Genomics Viewer (IGV). For ChIP-qPCR validation, purified DNA fragments were analyzed by quantitative PCR using primers spanning the ESRRG promoter region identified by ChIP-seq analysis. ChIP-qPCR enrichment was calculated as the percentage of input DNA. Normal IgG was included as a negative immunoprecipitation control. Primer sequences are provided in Supplementary Table S3.

### Dual-luciferase reporter assay

We used a dual luciferase reporter gene assay to evaluate the regulatory effect of ESRRG on the transcriptional activity of CDK1 promoter. A potential ERRγ-binding site within the CDK1 promoter was predicted using the hTFtarget database. Wild-type and mutant CDK1 promoter fragments containing the predicted site were synthesized and cloned into the pGL3-Basic luciferase reporter vector. HEK293T cells were cotransfected with the wild-type or mutant reporter construct, the pcDNA3.1-ESRRG expression plasmid or corresponding empty vector, and a Renilla luciferase control plasmid. After 48 h, firefly and Renilla luciferase activities were measured using the Dual-Luciferase® Reporter Assay System (Promega, E1910). Firefly luciferase activity was normalized to Renilla luciferase activity. In parallel, the activities of the WT and Mut constructs under ESRRG overexpression were compared to validate the functional relevance of the predicted binding site.

### Generation of conditional knockout mice

Conditional knockout mice were generated using the Cre-loxP system. Pten^f/f^ mice (B6.129S4-Ptentm1Hwu/J, JAX stock no. 006440) and Pgr-Cre mice (B6.129S(Cg)-Pgrtm1.1(cre)Shah/AndJ, JAX stock no. 017915) were purchased from The Jackson Laboratory. To generate uterine-specific Pten conditional knockout mice, Pten^f/f^ mice were crossed with Pgr-Cre mice. Mice carrying homozygous floxed Pten alleles and the Pgr-Cre transgene were used as conditional knockout mice, whereas Cre-negative littermate Pten^f/f^ mice were used as controls. Genomic DNA was extracted from tail biopsies, and genotyping was performed by PCR using primers specific for the floxed Pten allele and the Pgr-Cre transgene. Mice were maintained under specific pathogen-free conditions with free access to food and water.

### Animal experiments

All animal experiments were conducted in accordance with the guidelines and protocols approved by the Committee on Animal Care at Tongji University (approval no. TJBG04026101). Female athymic BALB/c nude mice, aged six weeks, were obtained from Shanghai Model Organisms and housed in a sterile environment. For the xenograft tumor growth assay, 6×10^6^ cells were subcutaneously injected into the flanks of the mice. After 16 days, the mice were euthanized, and tumor volumes were measured. Tumor volume was calculated using the formula: Tumor volume (V) = length × width^2^ × 0.5 (mm^3^). Additionally, tumors were harvested for histological examination. For *in vivo* drug treatment, mice bearing established xenograft tumors were randomly assigned to three groups: vehicle, QC6352 at 25 mg/kg, and QC6352 at 50 mg/kg (n = 5 per group). Mice received intraperitoneal injections of vehicle or QC6352 every other day for 16 days. The mice were then sacrificed, and the tumors were removed, weighed, and fixed for the IHC examination.

### Statistical analysis

Data are expressed as mean ± SD or mean ± SEM, derived from at least three independent experiments, as described in the figure legends. The sample sizes were determined based on previous experience with similar experiments and reference to relevant published studies. To compare two groups, a two-tailed Student's t-test was used, while for multiple group comparisons, one-way analysis of variance (ANOVA) followed by Tukey's post hoc test was applied. Survival analysis was conducted using the Kaplan-Meier method, with statistical differences assessed by the log-rank test. Spearman's correlation coefficient was used to examine the relationships between the expression levels of KDM4A, ERRγ, and CDK1. All statistical analyses were performed with GraphPad Prism 8.0 software, and a p-value of less than 0.05 was considered statistically significant.

## Results

### High KDM4A expression is associated with poor prognosis in UCEC patients

To systematically analyze the expression profile of the histone demethylase (KDM) family in EC, we conducted a comprehensive analysis of the mRNA expression levels of all 24 members based on the Cancer Genome Atlas (TCGA) and Gene Expression Omnibus (GEO) databases. By integrating data from TCGA and GEO, we analyzed the mRNA expression differences of all 24 KDM family members in tumor and adjacent normal tissues, as shown in Figure 1A. The Venn diagram in Figure 1B shows five differentially expressed genes shared between the two datasets. Kaplan-Meier survival analysis of these five differentially expressed genes revealed that only the expression level of KDM4A was significantly correlated with the prognosis of UCEC patients, with its high expression significantly associated with poor overall survival (Figure 1C and Fig. S1A-D). Analysis of UCEC samples in the TCGA showed that the expression level of KDM4A in tumor tissues (n=530) was significantly higher than in normal tissues (n=34) (Fig. 1D). Figure 1E shows that similar findings were noted in cancer tissues and their matched non-cancerous counterparts. Moreover, elevated mRNA expression levels of KDM4A in UCEC were confirmed using two separate GEO datasets: GSE17025 and GSE106191 (Fig. 1F). Interrogation of the CPTAC database revealed that KDM4A protein expression is upregulated in endometrial carcinoma relative to normal tissue (Figure 1G-H). Furthermore, Western blot (Figure 1I) and IHC analysis (Figure 1J) demonstrated markedly elevated KDM4A protein levels in tumor tissues compared with normal tissues. Notably, KDM4A expression was significantly higher in patients with copy number amplification or gain than in those with shallow deletion or diploid status (Fig. 1K). KDM4A expression was significantly associated with copy-number status, suggesting that copy-number alterations may contribute to elevated KDM4A expression in a subset of UCEC samples. CPTAC proteomic analysis showed that KDM4A protein expression was significantly higher in UCEC tissues than in normal endometrial tissues across multiple clinical stages (Figure S1E). Moreover, KDM4A expression increased progressively with histological grade, with the highest level observed in grade 3 tumors (Figure S1F). These findings indicate that KDM4A is upregulated in UCEC and is associated with poor tumor differentiation. The ROC curve analysis showed that KDM4A had good diagnostic discrimination capacity for distinguishing EC tissues from normal tissues, with an AUC of 0.887 (95% CI: 0.847-0.922) (Fig. S1G). KDM4A showed the ability to discriminate tumor from normal tissue in the analyzed TCGA cohort; however, its diagnostic utility requires validation in independent clinical cohorts. Clinicopathological characteristics stratified by KDM4A expression are summarized in Supplementary Table S4.

### KDM4A promotes EC cell proliferation, migration, and invasion

In tumor research, the influence of genes on the proliferation and metastatic ability of tumor cells is a key factor that must be considered 29, 30. The analysis of UCEC data from GEPIA revealed significant correlations between KDM4A expression and the expression of SNAI1, MKI67, VIM, and CDH2 (Fig. S2A-D). Given the upregulation of KDM4A in EC tissues, we subsequently assessed its impact on the proliferation, migration, and invasive potential of EC cells. We used three shRNAs to knock down KDM4A expression in EC cells and evaluated the knockdown efficiency (Figure 2A). We identified sh-KDM4A#1 and #2 as the most efficient for subsequent experiments. CCK-8 and EdU assays demonstrated that, compared to the control group, knockdown of KDM4A significantly inhibited cell proliferation in Ishikawa cells (Figure 2B-D). Transwell migration and invasion assays demonstrated that, relative to the control group, KDM4A knockdown significantly reduced cell migration and invasion (Figure 2E-F). The migration and invasion of cancer cells are closely associated with epithelial-mesenchymal transition (EMT), and alterations in the expression of key molecular proteins within the EMT signaling pathway were assessed via Western blot analysis 31-33. The results demonstrated that KDM4A knockdown led to a significant upregulation of the epithelial marker E-Cadherin, while the expression of mesenchymal markers N-Cadherin and Vimentin was notably downregulated (Figure 2G-H). In contrast, we established an overexpression model of KDM4A in HEC1-B cells (Figure 2I). Overexpression of KDM4A significantly enhanced the proliferation potential of HEC1-B cells (Figure 2J-L). Moreover, the overexpression of KDM4A markedly augmented the cell migration and invasion (Figure 2M-N). Upon overexpression of KDM4A, a significant downregulation of E-Cadherin was observed, accompanied by a notable upregulation of N-Cadherin and Vimentin (Figure 2O-P). To further investigate whether KDM4A regulates cell-cycle progression, cell-cycle distribution was analyzed by flow cytometry. In Ishikawa cells, knockdown of KDM4A using two independent shRNAs significantly increased the proportion of cells in the G2/M phase compared with the sh-Ctrl group (Figure S2E-F). Changes in the S-phase population were relatively modest and were not completely consistent between the two shRNAs. These results indicate that KDM4A depletion induces the accumulation of EC cells in the G2/M phase. Conversely, KDM4A overexpression in HEC1-B cells significantly reduced the proportion of cells in the G2/M phase, whereas no significant difference was observed in the S-phase population (Figure S2G-H). Collectively, these findings support the involvement of KDM4A in the regulation of G2/M-phase progression in EC cells.

### KDM4A regulates ERRγ expression by modulating H3K9me3 enrichment at the ESRRG promoter

Previous studies have established that KDM4A is a crucial histone demethylase, playing a pivotal role in the onset and progression of tumors through its epigenetic regulatory functions 34-36. KDM4A possesses histone demethylation activity, targeting both H3K9me3 and H3K36me3 37-39. Western blot analysis revealed that knockdown of KDM4A significantly increased the level of H3K9me3 modification (Figure 3A-B), while exerting no significant effect on H3K36me3 levels (Fig. S3A-D). Conversely, overexpression of KDM4A markedly reduced H3K9me3 levels (Figure 3C-D). Collectively, these findings suggest that H3K9me3 is more responsive than H3K36me3 to KDM4A manipulation under the conditions examined. Given its role in regulating target gene transcription through demethylation, this study employed ChIP-seq to screen for potential target genes of KDM4A. Figure 3E shows the overlap among genes exhibiting altered H3K9me3 enrichment following KDM4A overexpression, genes correlated with KDM4A expression, and differentially expressed genes in TCGA-UCEC. Four genes—ESRRG, MYCBP2, TAF1, and CSNK1G1—were shared across all three datasets. Figure S4A shows the ChIP-seq signal intensity trajectory for the ESRRG gene region. The results indicate that, near the transcription start site of the ESRRG gene, the immunoprecipitation samples from the overexpression group (OE_2IP) exhibited visibly lower normalized H3K9me3 signal intensity compared to the control group (NC_2IP), particularly in the red-highlighted area. No comparable alteration in H3K9me3 enrichment was observed at the MYCBP2, TAF1, or CSNK1G1 loci (Figure S4B-D). GEPIA database analysis demonstrated a positive correlation trend between KDM4A and ESRRG in EC (Figure 3F). RT-qPCR and western blot analyses showed that ESRRG expression changed in parallel with KDM4A manipulation. Specifically, KDM4A knockdown in Ishikawa cells significantly decreased ESRRG mRNA and protein levels (Figure 3G-I), whereas its overexpression in HEC1-B cells markedly increased them (Figure 3J-L). To elucidate the underlying mechanism, ChIP-qPCR assays revealed that KDM4A depletion in Ishikawa cells led to an increase in H3K9me3 enrichment at the ESRRG promoter. Conversely, KDM4A overexpression in HEC1-B cells reduced this repressive histone mark (Figure 3M-N). These findings support a model in which KDM4A facilitates ESRRG transcription by reducing the enrichment of the repressive H3K9me3 modification at its promoter. However, because KDM4A-specific ChIP analysis was not performed, direct occupancy of KDM4A at the ESRRG promoter cannot be concluded from the present data. To further investigate this relationship *in vivo*, we utilized the Pten^f/f^ Pgr-cre mouse model, which spontaneously develops EC (Figure S5A-B). IHC analysis of tumor specimens from both this murine model and human EC patients revealed a significant positive correlation between KDM4A and ERRγ protein levels (Figure S5C-D). Furthermore, mIHC images show that KDM4A knockdown in Ishikawa cells increased H3K9me3 and reduced KDM4A expression, while KDM4A overexpression in HEC1-B cells decreased H3K9me3 and enhanced ERRγ expression. These findings suggest that KDM4A may promote ESRRG transcription, at least in part, by reducing H3K9me3 enrichment at the ESRRG promoter; however, direct occupancy of KDM4A at this locus remains to be established (Figure S6).

### ERRγ promotes EC cell-cycle progression in association with the Cyclin B1/CDK1 axis

By analyzing the UALCAN database, we found that the mRNA expression level of ESRRG in EC is higher than that in normal tissues, suggesting a potential association between ERRγ expression and EC (Figure 4A). The expression of ERRγ protein was found to be increased in tumors compared to normal tissues, as shown by Western blot analysis of human EC samples (Figure 4B). Based on the expression level of ESRRG in TCGA-UCEC, the samples were divided into high-expression and low-expression groups according to the median, and differential expression analysis was performed. The results of differentially expressed genes are shown in Figure 4C. KEGG enrichment analysis suggests that cell cycle-related pathways are significantly affected (FDR < 0.05) (Figure 4D). To further elucidate the functional role of ERRγ in EC, we constructed cell models with stable overexpression or knockdown of ESRRG in HEC1-B and Ishikawa cell lines. Our analysis of mRNA and protein levels showed that ERRγ was notably reduced in HEC1-B cells compared to the control group (Figure S7A-B). Cell cycle analysis by flow cytometry showed that ERRγ knockdown induced G2/M phase arrest, as evidenced by an increased proportion of cells in this phase (Figure 4E-F). In contrast, we constructed a stable cell line overexpressing ERRγ in Ishikawa cells (Figure S7A-B). Flow cytometry analysis revealed that overexpression of ERRγ in Ishikawa cells resulted in a significant shift in cell cycle distribution, marked by a concomitant reduction in the G2/M phase population (Figure 4G-H). Western blotting and densitometric analyses showed that ERRγ knockdown significantly reduced Cyclin B1 and CDK1 protein levels in HEC1-B cells (Figure 4I-J), whereas ERRγ overexpression increased the expression of both proteins in Ishikawa cells (Figure 4K-L). These reciprocal changes support a positive regulatory relationship between ERRγ and the G2/M-associated cell-cycle regulators Cyclin B1 and CDK1. To elucidate the potential role of ERRγ as a transcription factor in the cell cycle progression of EC cells, we employed the hTFtarget database for in silico prediction. This analysis revealed a potential binding site for ERRγ within the promoter region of the CDK1 gene (Figure 4M). Furthermore, CDK1 mRNA expression was suppressed upon ERRγ silencing and elevated following ERRγ overexpression (Figure 4N-O). Dual luciferase assay results showed that ERRγ overexpression significantly increased luciferase activity driven by the wild-type CDK1 promoter, whereas it had no significant effect on the mutant promoter (Figure 4P). Additionally, correlation analysis using the GEPIA database revealed positive correlations between the expression levels of KDM4A, ESRRG, and CDK1 in TCGA-UCEC patients (Figure S7C-D).

### ERRγ partially mediates the effects of KDM4A on EC cell-cycle progression

To investigate whether ERRγ contributes to the cellular effects associated with KDM4A, we generated two stable cell lines: one with KDM4A knockdown and ERRγ overexpression (sh-KDM4A + OE-ERRγ) (Figure 5A-B) and the other with KDM4A overexpression and ERRγ knockdown (OE-KDM4A + sh-ERRγ) (Figure 5C-D). Western blot results confirmed that both stable cell lines were successfully established. Colony formation and CCK-8 assays showed that KDM4A knockdown significantly reduced the proliferative capacity of Ishikawa cells, whereas ectopic ERRγ expression partially restored cell proliferation in KDM4A-depleted cells (Figure 5E-F, I). Conversely, KDM4A overexpression enhanced the proliferation of HEC1-B cells, and this effect was partially attenuated by ERRγ knockdown (Figure 5G-H, K). Consistent with these findings, KDM4A depletion induced alterations in cell-cycle distribution in Ishikawa cells, including G2/M-phase accumulation, which were partially reversed by ERRγ overexpression (Figure 5J). In HEC1-B cells, ERRγ knockdown partially reversed the changes in cell-cycle distribution induced by KDM4A overexpression (Figure 5L). These findings support a functional role for ERRγ as a downstream mediator of KDM4A-associated cell-cycle regulation. Western blot analysis further showed that KDM4A knockdown decreased Cyclin B1 and CDK1 expression in Ishikawa cells, whereas ERRγ overexpression partially restored their expression in KDM4A-depleted cells (Figure 5M-N). Conversely, KDM4A overexpression increased Cyclin B1 and CDK1 expression in HEC1-B cells, and these increases were partially attenuated by ERRγ knockdown (Figure 5O-P). Together, these results suggest that ERRγ contributes to KDM4A-mediated regulation of the Cyclin B1/CDK1 axis. In addition, KDM4A knockdown significantly impaired the migration and invasion of Ishikawa cells, whereas ERRγ overexpression partially restored these phenotypes. The migratory and invasive capacities of the sh-KDM4A + OE-ERRγ group were significantly greater than those of the sh-KDM4A + NC-ERRγ group, although the rescue was incomplete relative to the control group. In HEC1-B cells, KDM4A overexpression promoted migration and invasion, whereas ERRγ knockdown partially attenuated these effects (Figure S8A-B). Collectively, these findings support the interpretation that ERRγ functions as an important, but potentially not exclusive, downstream mediator of KDM4A-associated proliferation, cell-cycle progression, migration, and invasion in EC cells.

### QC6352 suppresses EC cell proliferation, migration, and invasion accompanied by reduced KDM4A and ERRγ expression

Multiple studies have suggested that QC6352 is a small-molecule inhibitor of Jumonji C domain-containing histone demethylases, including members of the KDM4 family 36, 40, 41. Preliminary findings highlight the significant impact of KDM4A on EC cell proliferation, leading to the hypothesis that small molecule inhibitors targeting KDM4 may offer a promising therapeutic strategy for EC patients. To examine whether QC6352 treatment is associated with changes in the KDM4A/ERRγ axis in EC cells, we treated HEC1-B and Ishikawa cells with varying concentrations of QC6352 (0, 50, 100 and 200 nM) for 48 hours 36, 41. As shown in Figures 6A-B, QC6352 treatment significantly reduced the expression of KDM4A and ERRγ in both cell lines. In the colony formation assay, QC6352 treatment markedly decreased the clonogenic potential of both Ishikawa and HEC1-B cells (Figures 6C-E). Cell proliferation assays revealed that QC6352 significantly inhibited cell growth in both cell lines, with the 100nM concentration showing the most pronounced inhibitory effect on cell viability (Figures 6F-G). QC6352 treatment significantly increased the proportion of cells in the G2/M phase, indicating G2/M-phase accumulation or arrest (Figures 6H and J). Western blot analysis further confirmed a reduction in Cyclin B1 and CDK1 expression following QC6352 treatment, suggesting downregulation of key regulators of the cell cycle (Figures 6I and K). Additionally, QC6352 treatment significantly inhibited the migration and invasion capabilities of both Ishikawa and HEC1-B cells (Figures 6L-M). In summary, these findings show that QC6352 suppresses EC cell proliferation, migration, and invasion and alters cell-cycle distribution, accompanied by reduced expression of KDM4A, ERRγ, Cyclin B1, and CDK1.

### KDM4A knockdown and QC6352 treatment suppress tumor growth *in vivo*

To investigate the role of KDM4A *in vivo* in EC progression, we established a subcutaneous tumor model in nude mice using sh-Ctrl and sh-KDM4A HEC1-B cells (Figure 7A). As shown in Figure 7B, knockdown of KDM4A (sh-KDM4A) significantly inhibited tumor growth in the mouse model. Both tumor volume (Figure 7C) and tumor weight (Figure 7D) were markedly greater in the sh-Ctrl group compared to the sh-KDM4A group. Additionally, IHC staining revealed a significant decrease in Ki67 expression in the sh-KDM4A group, indicating reduced cell proliferation (Figures 7E-F). To further evaluate the antitumor activity of QC6352 *in vivo*, we administered QC6352 at doses of 0, 25, and 50 mg/kg to the mouse models (Figure 7G). As demonstrated in Figure 7H, intraperitoneal administration of QC6352 significantly suppressed tumor growth in the mouse model. Tumor volume in the QC6352 treatment group was significantly reduced compared to the control group, and tumor growth was markedly inhibited across different concentrations (Figure 7I-J). IHC analysis revealed that Ki67 expression was significantly reduced in the QC6352 treatment group compared to the control group, suggesting that reduced Ki67 staining was consistent with decreased tumor-cell proliferation following QC6352 treatment (Figure 7K-L). These findings collectively indicate that QC6352 effectively suppresses tumor growth in a mouse model of EC.

## Discussion

This study characterizes the association of the KDM4A-ERRγ-CDK1 axis with malignant phenotypes and cell-cycle regulation in EC (Figure 8). We demonstrate that KDM4A promotes ERRγ expression in association with reduced H3K9me3 enrichment at the ESRRG promoter, suggesting attenuation of H3K9me3-associated repression. However, the present H3K9me3 ChIP data do not establish direct occupancy of KDM4A at this locus. Increased ERRγ expression was associated with altered G2/M-phase distribution, increased Cyclin B1/CDK1 expression, and enhanced EC cell proliferation. These epigenetic changes were associated with enhanced EC cell proliferation, migration, and invasion. Notably, the KDM4 inhibitor QC6352 exerted antitumor effects *in vitro* and *in vivo*, accompanied by reduced expression of components of the KDM4A/ERRγ-associated cell-cycle axis. These findings support further investigation of the KDM4A/ERRγ-associated axis as a potential therapeutic vulnerability in EC.

Targeting epigenetic regulators, such as histone modification pathways, offers a promising strategy to disrupt tumor progression, positioning epigenetic regulation as a key focus for EC treatment 42-44. As a critical member of the histone demethylase family, KDM4A plays a complex role in cancer development and holds significant potential as a therapeutic target 45-47. Elevated KDM4A expression in colorectal cancer, bladder cancer, and other solid tumors is strongly associated with poor prognosis 48-50. Research has shown that KDM4A promotes cancer cell proliferation, migration, invasion, and drug resistance 38, 51, 52. Inhibiting KDM4A induces cellular senescence, increases cell death, and enhances immune responses, further supporting its therapeutic potential 37, 53, 54. Current studies are not only elucidating the mechanisms by which KDM4A drives tumorigenesis but also exploring small molecule inhibitors targeting KDM4A, offering new avenues for precision cancer therapy 55, 56. Our findings identify an ERRγ-associated cell-cycle regulatory pathway that may contribute to the oncogenic effects of KDM4A in EC. While previous studies have shown that certain histone-modifying enzymes promote tumor progression by regulating pathways such as PI3K/AKT or Wnt/β-catenin signaling, these findings provide evidence for an epigenetic cell-cycle regulatory mechanism involving KDM4A and ERRγ in EC 57, 58. Collectively, these results highlight a complex epigenetic regulatory network in EC and provide a novel theoretical foundation for precision therapeutic strategies targeting KDM4A.

Although this study characterizes the oncogenic role of KDM4A in EC and demonstrates the antitumor activity of QC6352 in cell-based and subcutaneous xenograft models, several limitations should be acknowledged. First, regarding experimental models, this study primarily relied on *in vitro* cell lines and subcutaneous xenograft models, which are commonly used in EC research. Although consistent anti-tumor effects were observed in both *in vitro* and *in vivo* experiments, subcutaneous xenograft models cannot fully recapitulate the native endometrial microenvironment, including hormone-dependent characteristics, stromal interactions, and immune components that critically influence tumor progression. Recent studies have highlighted the essential roles of the tumor microenvironment, immune cell infiltration, and metabolic reprogramming in EC development. Therefore, further validation of the biological function of the KDM4A-ERRγ axis in orthotopic models, patient-derived xenograft (PDX) models, or immunocompetent systems is warranted. Second, at the mechanistic level, although our findings suggest that KDM4A promotes ERRγ expression in association with reduced H3K9me3 enrichment at the ESRRG promoter, KDM4A is a chromatin-modifying enzyme with broad regulatory functions. Previous studies have shown that KDM4A participates in DNA damage repair, metabolic regulation, and EMT-related gene regulation in various malignancies. Thus, it remains unclear whether additional critical downstream targets of KDM4A exist in EC beyond ERRγ. Comprehensive multi-omics approaches would be valuable to delineate the global transcriptional network regulated by KDM4A. Third, from a pharmacological perspective, QC6352 is a KDM4-family inhibitor with activity against KDM4A, KDM4B, KDM4C, and KDM4D, rather than a KDM4A-specific inhibitor. Although QC6352 significantly reduced KDM4A/ERRγ expression and suppressed tumor growth in our models, potential off-target effects on other KDM family members cannot be excluded. The development and application of more selective KDM4A inhibitors will be essential to further validate the therapeutic relevance of this signaling axis. Finally, at the clinical translational level, studies investigating KDM4A in EC remain limited and are largely confined to expression profiling and *in vitro* functional analyses. The present study did not systematically assess the association of the KDM4A-ERRγ-CDK1 axis with molecular subtypes, tumor stage, recurrence risk, or treatment response using large-scale clinical cohorts, TCGA datasets, or long-term follow-up data. Consequently, the prognostic and predictive value of this signaling axis requires further clinical validation. In summary, future studies integrating multi-omics analyses, orthotopic and immunocompetent animal models, and large-scale clinical datasets are needed to further clarify the role of the KDM4A-ERRγ-CDK1 axis in EC progression and to facilitate its translation into precision epigenetic therapeutic strategies.

## Conclusions

Our findings show that KDM4A is upregulated in EC and is associated with enhanced cell proliferation, migration, and invasion. Manipulation of KDM4A expression altered H3K9me3 enrichment at the ESRRG promoter and produced corresponding changes in ERRγ expression, supporting a model in which KDM4A may facilitate ESRRG transcription by attenuating H3K9me3-associated repression. ERRγ was associated with increased CDK1 transcription, elevated Cyclin B1 and CDK1 expression, and altered G2/M-phase distribution. Rescue experiments further suggested that ERRγ partially mediates the effects of KDM4A on malignant cellular phenotypes and cell-cycle regulation. In addition, QC6352 suppressed EC cell growth *in vitro* and reduced xenograft tumor growth *in vivo*, accompanied by downregulation of the KDM4A/ERRγ-associated cell-cycle axis. Collectively, these findings support further investigation of the KDM4A-H3K9me3-ERRγ regulatory relationship and KDM4-family inhibition in EC.

## Supplementary Material

Supplementary figures and tables.

## Figures and Tables

**Figure 1:**
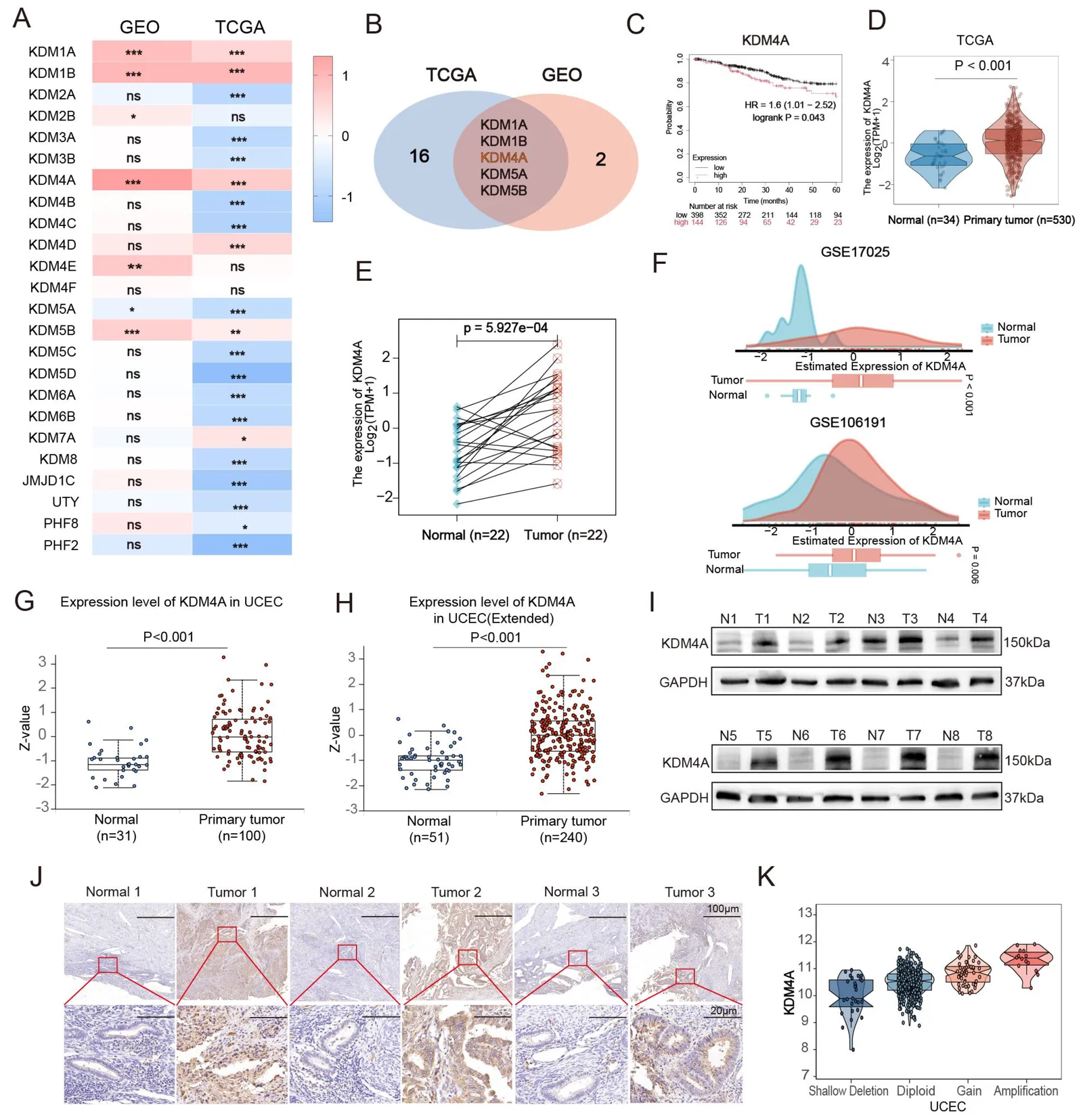
** KDM4A is identified as an EC-associated histone lysine demethylase that is upregulated in UCEC and associated with poor overall survival. A.** Analysis of mRNA expression levels of 24 histone lysine demethylases in normal and EC tissues using the GEO (GSE17025) and TCGA databases.** B.** The intersection genes of mRNA differential expression of 24 histone lysine demethylases in GEO (GSE17025) and TCGA databases. **C.** Kaplan-Meier analysis of overall survival (OS) rates among UCEC patients based on high or low KDM4A expression. **D.** TCGA database analysis of KDM4A mRNA expression in UCEC and normal tissues. **E.** KDM4A expression was analyzed in 22 paired UCEC patient samples obtained from the TCGA database. **F.** Data from the GSE17025 and GSE106191 databases were explored to assess the expression of KDM4A in normal and EC tissues. **G-H.** The protein levels of KDM4A in normal and EC tissues were assessed using data from the CPTAC databases. **I.** Western blot analysis of KDM4A expression in EC tumor tissues (T) and adjacent normal tissues (N). **J.** Immunohistochemistry (IHC) staining of KDM4A in EC tumor tissues and adjacent normal tissues. Scale bars, 100 μm and 20 μm, respectively. **K.** KDM4A gene copy number gain and amplification were positively associated with increased mRNA expression in the TCGA-UCEC cohort. *P < 0.05; **P < 0.01; ***P < 0.001.

**Figure 2:**
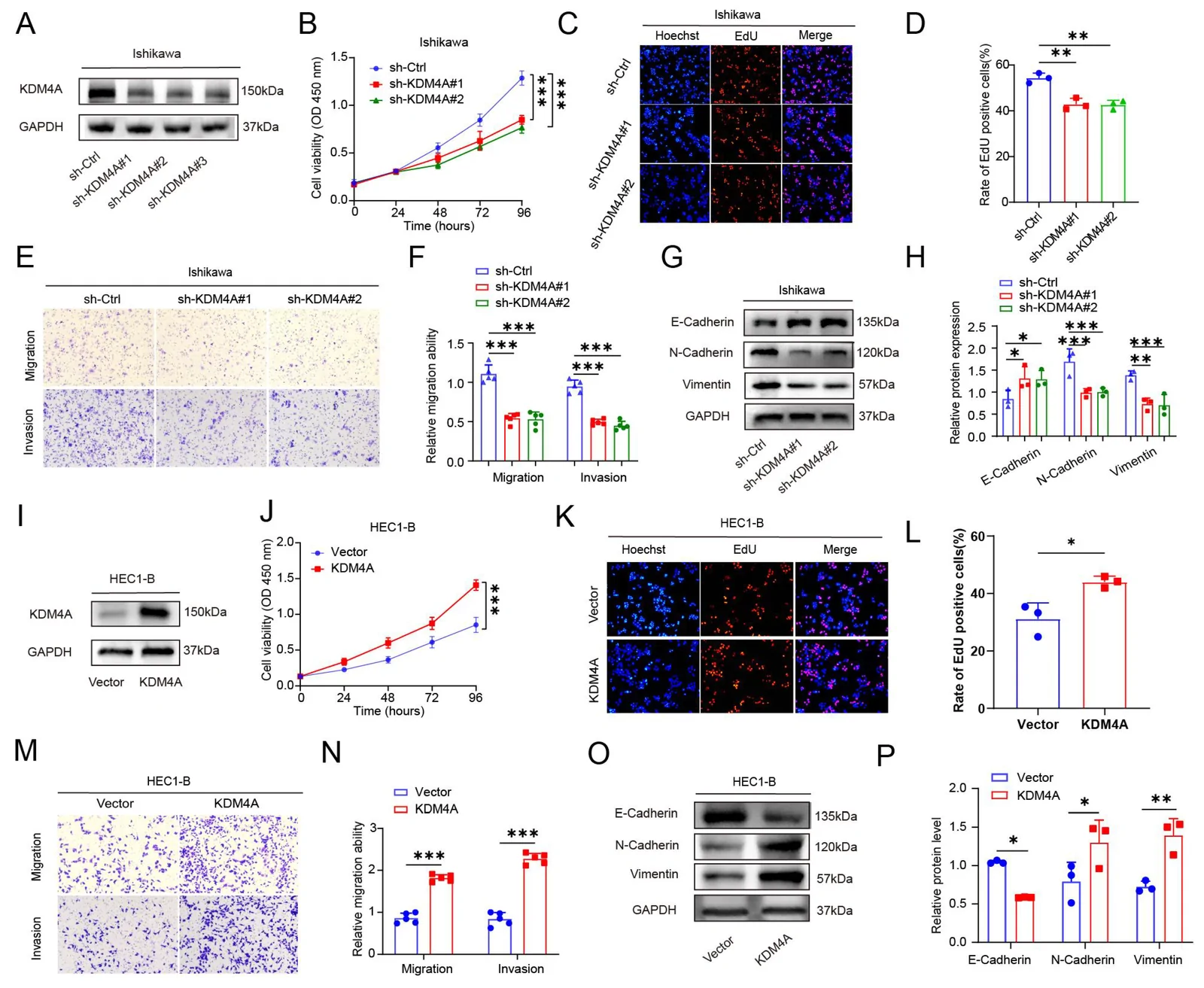
** KDM4A regulates EC cell proliferation, migration, and invasion *in vitro*. A.** Western blot analysis confirmed the knockdown efficiency of KDM4A in Ishikawa cells.** B-D.** CCK-8 and EdU assays revealed that sh-KDM4A significantly inhibited cell growth *in vitro*.** E-F.** Transwell assays demonstrated that downregulation of KDM4A significantly reduced the migratory and invasive capabilities of Ishikawa cells.** G-H.** Western blot analysis revealed the levels of epithelial-mesenchymal transition (EMT)-related proteins, including E-cadherin, N-cadherin, and Vimentin.** I.** Western blot analysis confirmed the efficient overexpression of KDM4A in HEC1-B cells.** J-L.** CCK-8 and EdU assays revealed that KDM4A overexpression significantly enhanced cell growth *in vitro*. **M-N.** Transwell assays demonstrated that KDM4A overexpression significantly enhanced the migration and invasion of HEC1-B cells.** O-P.** Western blot analysis revealed that KDM4A overexpression significantly decreased the level of E-cadherin, while increasing the levels of N-cadherin and Vimentin in HEC1-B cells. *P < 0.05; **P < 0.01; ***P < 0.001.

**Figure 3:**
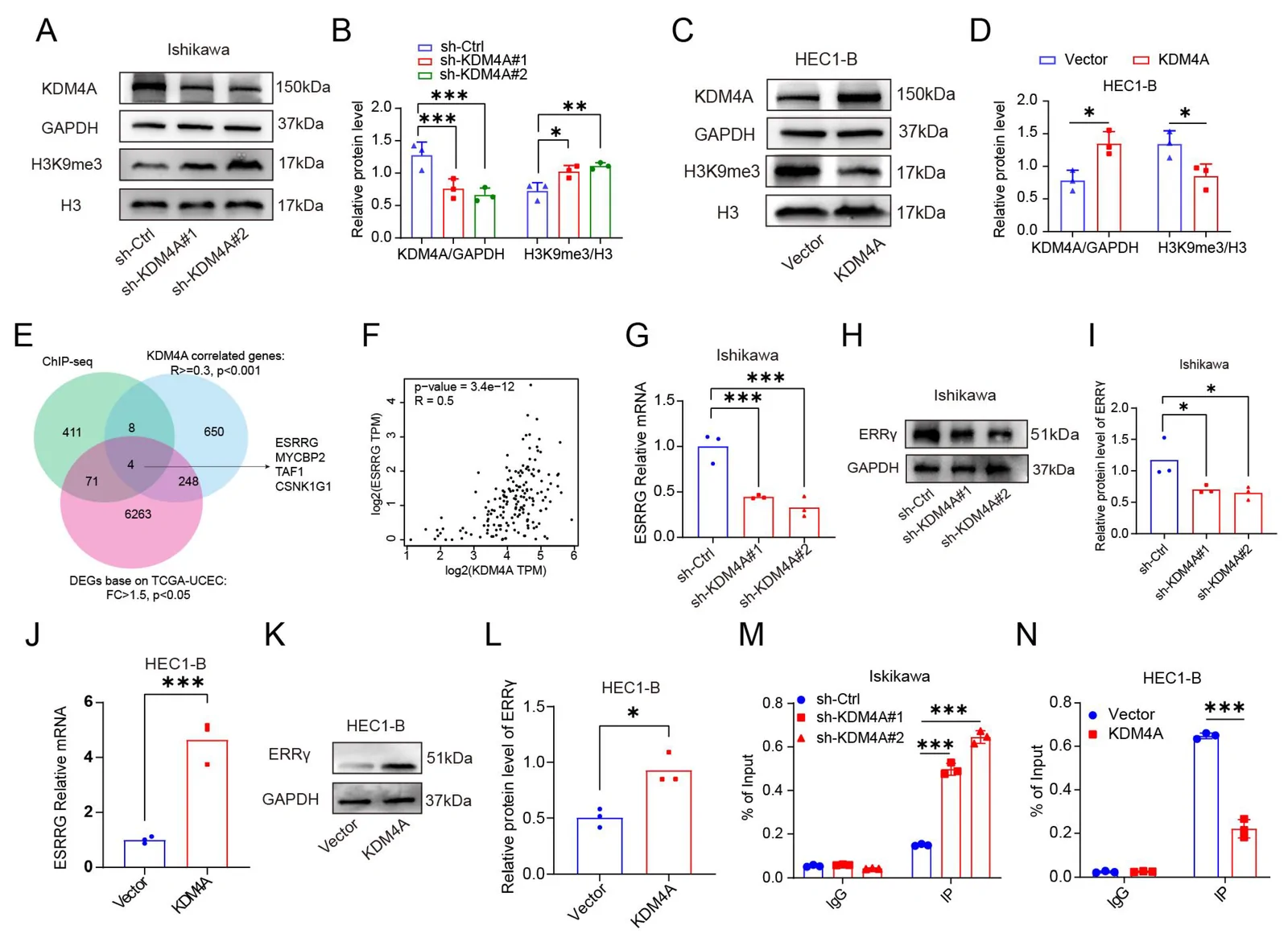
** KDM4A regulates ERRγ expression by modulating H3K9me3 enrichment at the ESRRG promoter. A-D.** Western blot analysis confirmed that KDM4A knockdown (A-B) and overexpression (C-D) altered H3K9me3 protein level. **E.** Venn diagram analysis revealed four overlapping genes associated with differential H3K9me3 enrichment, KDM4A-correlated genes, and differentially expressed genes in TCGA-UCEC.** F.** Correlation of KDM4A and ESRRG mRNA expression in the GEPIA database. **G-I.** qPCR and western blot analyses showed that KDM4A knockdown significantly decreased ESRRG expression. **J-L.** qPCR and western blot analyses demonstrated that KDM4A overexpression significantly upregulated ESRRG expression. **M-N.** Independent ChIP-qPCR assays were conducted to assess H3K9me3 enrichment in both KDM4A-overexpressing and KDM4A-knockdown cells.

**Figure 4:**
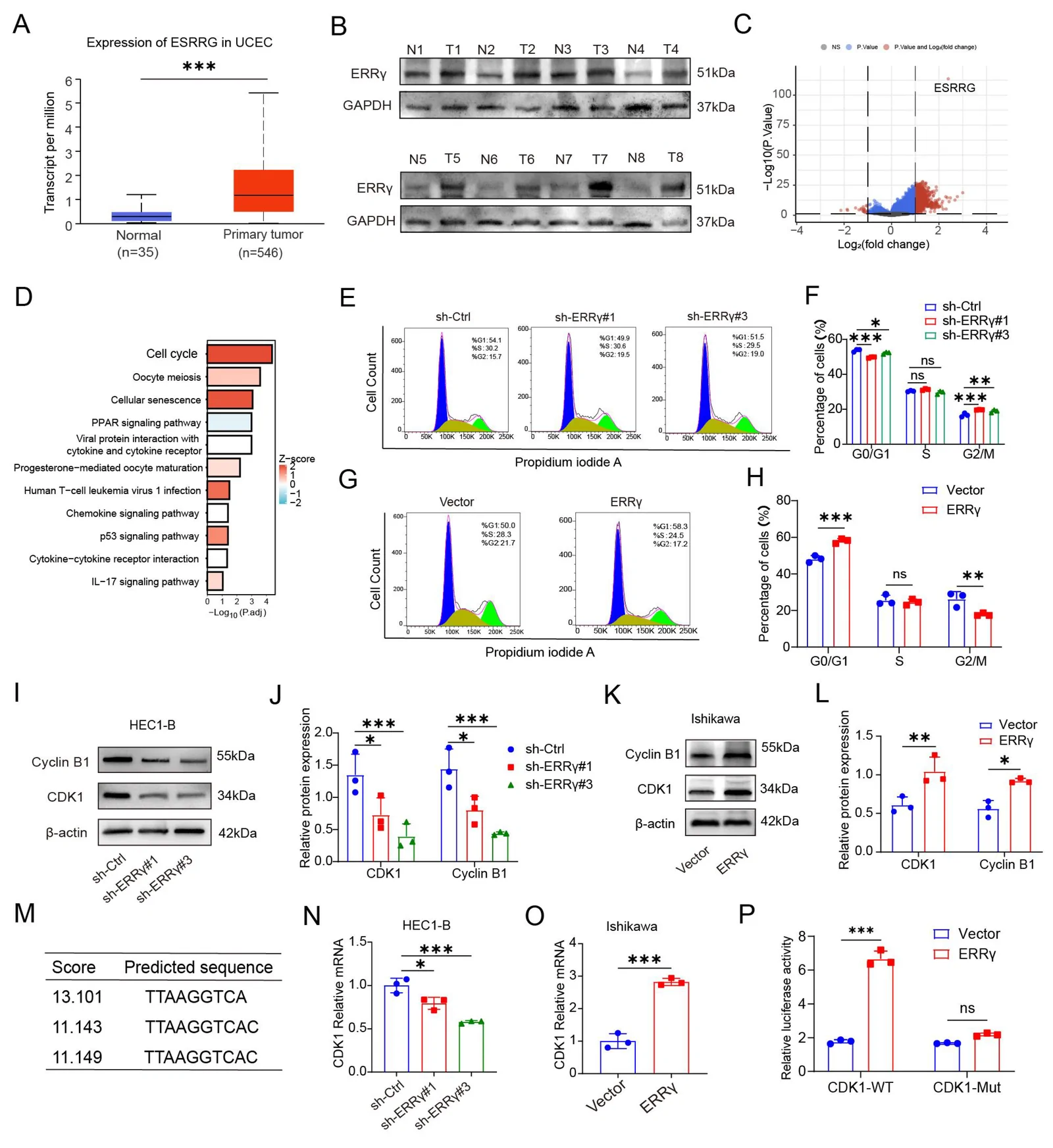
** ERRγ promotes EC cell-cycle progression in association with the Cyclin B1/CDK1 axis. A.** Relative RNA levels of ESRRG in UCEC tumor samples (n = 546) compared to normal samples (n = 35) from the UALCAN database. **B.** ERRγ expression in normal and EC tissues was detected by western blot analysis. **C.** Volcano plot showing differential expression genes between high and low ESRRG groups.** D.** KEGG analysis identified differentially enriched pathways between high and low ESRRG expression groups.** E-F.** Representative flow-cytometric profiles and quantitative analysis of cell-cycle distribution in HEC1-B cells transduced with sh-Ctrl and sh-ERRγ. **G-H.** Representative flow-cytometric profiles and quantitative analysis of cell-cycle distribution in Ishikawa cells transduced with the empty vector or an ERRγ-overexpression construct. **I-L.** Western blot analysis revealed that ERRγ knockdown decreased Cyclin B1 and CDK1 expression (I-J), whereas ERRγ overexpression increased their levels (K-L).** M.** hTFtarget database was employed to predict ERRγ-binding site within the CDK1 promoter sequence. **N-O.** CDK1 mRNA levels were assessed by qRT-PCR following ERRγ knockdown in HEC1-B cells (N) and ERRγ overexpression in Ishikawa cells (O). **P.** The activity of wild-type and mutant CDK1 promoters was assessed by dual-luciferase reporter assays in HEK293T cells overexpressing ERRγ. *P < 0.05; **P < 0.01; ***P < 0.001.

**Figure 5:**
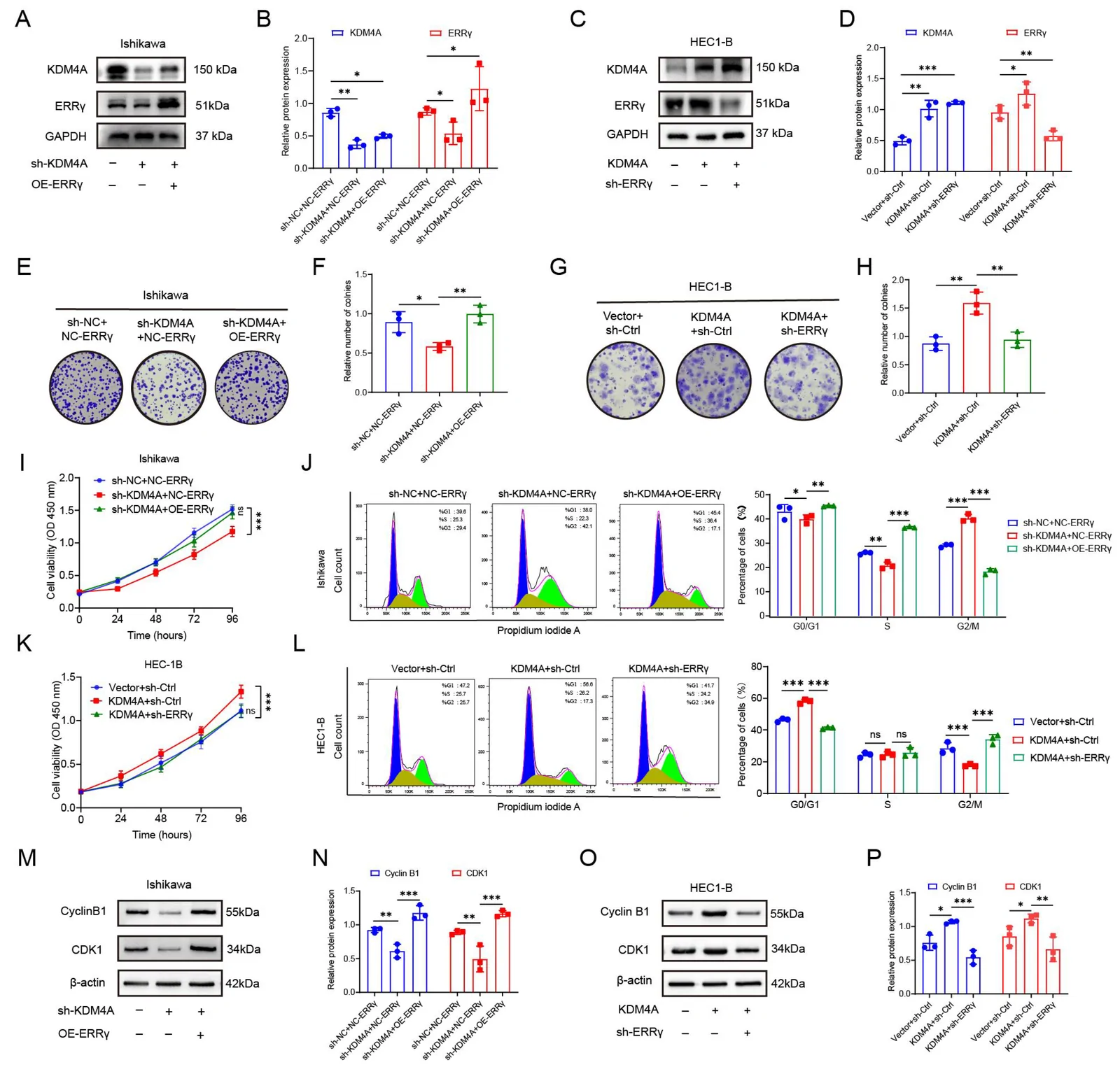
** ERRγ partially mediates the effects of KDM4A on EC cell-cycle progression. A-B.** Representative western blots and densitometric quantification of KDM4A and ERRγ expression in Ishikawa cells transduced with sh-NC + NC-ERRγ, sh-KDM4A + NC-ERRγ, or sh-KDM4A + OE-ERRγ. **C-D.** Representative western blots and densitometric quantification of KDM4A and ERRγ expression in HEC1-B cells transduced with Vector + sh-Ctrl, KDM4A + sh-Ctrl, or KDM4A + sh-ERRγ. **E-F.** Representative images and quantification of colony formation in Ishikawa cells with the indicated treatments. **G-H.** Representative images and quantification of colony formation in HEC1-B cells with the indicated treatments. **I.** CCK-8 analysis of cell proliferation in Ishikawa cells with the indicated treatments. **J.** Representative flow-cytometric profiles and quantification of cell-cycle distribution in Ishikawa cells with the indicated treatments. **K.** CCK-8 analysis of cell proliferation in HEC1-B cells with the indicated treatments. **L.** Representative flow-cytometric profiles and quantification of cell-cycle distribution in HEC1-B cells with the indicated treatments. **M-N.** Representative western blots and densitometric quantification of Cyclin B1 and CDK1 protein expression in Ishikawa cells with the indicated treatments. **O-P.** Representative western blots and densitometric quantification of Cyclin B1 and CDK1 protein expression in HEC1-B cells with the indicated treatments. Data are presented as the mean ± SD. ns, not significant; *P < 0.05; **P < 0.01; ***P < 0.001.

**Figure 6:**
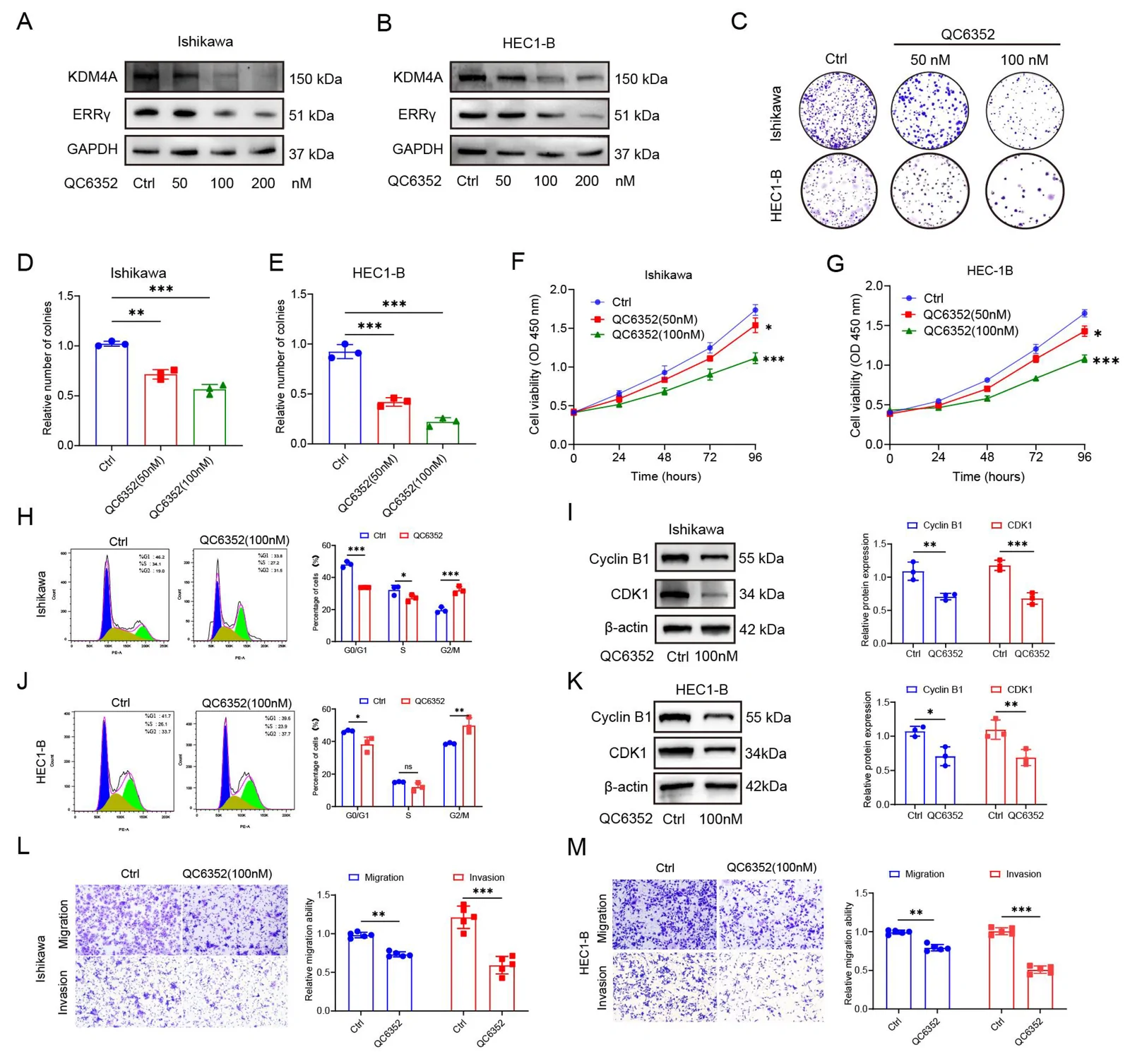
** QC6352 suppresses EC cell proliferation, migration, and invasion accompanied by reduced KDM4A and ERRγ expression. A-B.** Western blot analysis of KDM4A and ERRγ protein expression in Ishikawa (A) and HEC1-B (B) cells treated with the indicated concentrations of QC6352 (0, 50, 100, and 200 nM). **C-E.** Representative images (C) and quantitative analysis of colony formation in Ishikawa (D) and HEC1-B (E) cells treated with QC6352 at 0, 50, or 100 nM. **F-G.** CCK-8 assays showing the viability of Ishikawa (F) and HEC1-B (G) cells treated with QC6352 at 0, 50, or 100 nM. **H.** Representative flow-cytometric profiles and quantitative analysis of cell-cycle distribution in Ishikawa cells treated with vehicle control or 100 nM QC6352. **I.** Representative western blots and densitometric quantification of Cyclin B1 and CDK1 protein expression in Ishikawa cells treated with vehicle control or 100 nM QC6352. **J.** Representative flow-cytometric profiles and quantitative analysis of cell-cycle distribution in HEC1-B cells treated with vehicle control or 100 nM QC6352. **K.** Representative western blots and densitometric quantification of Cyclin B1 and CDK1 protein expression in HEC1-B cells treated with vehicle control or 100 nM QC6352. **L-M.** Representative images and quantitative analysis of Transwell migration and invasion assays in Ishikawa (L) and HEC1-B (M) cells treated with vehicle control or 100 nM QC6352. Data are presented as the mean ± SD. *P < 0.05; **P < 0.01; ***P < 0.001.

**Figure 7:**
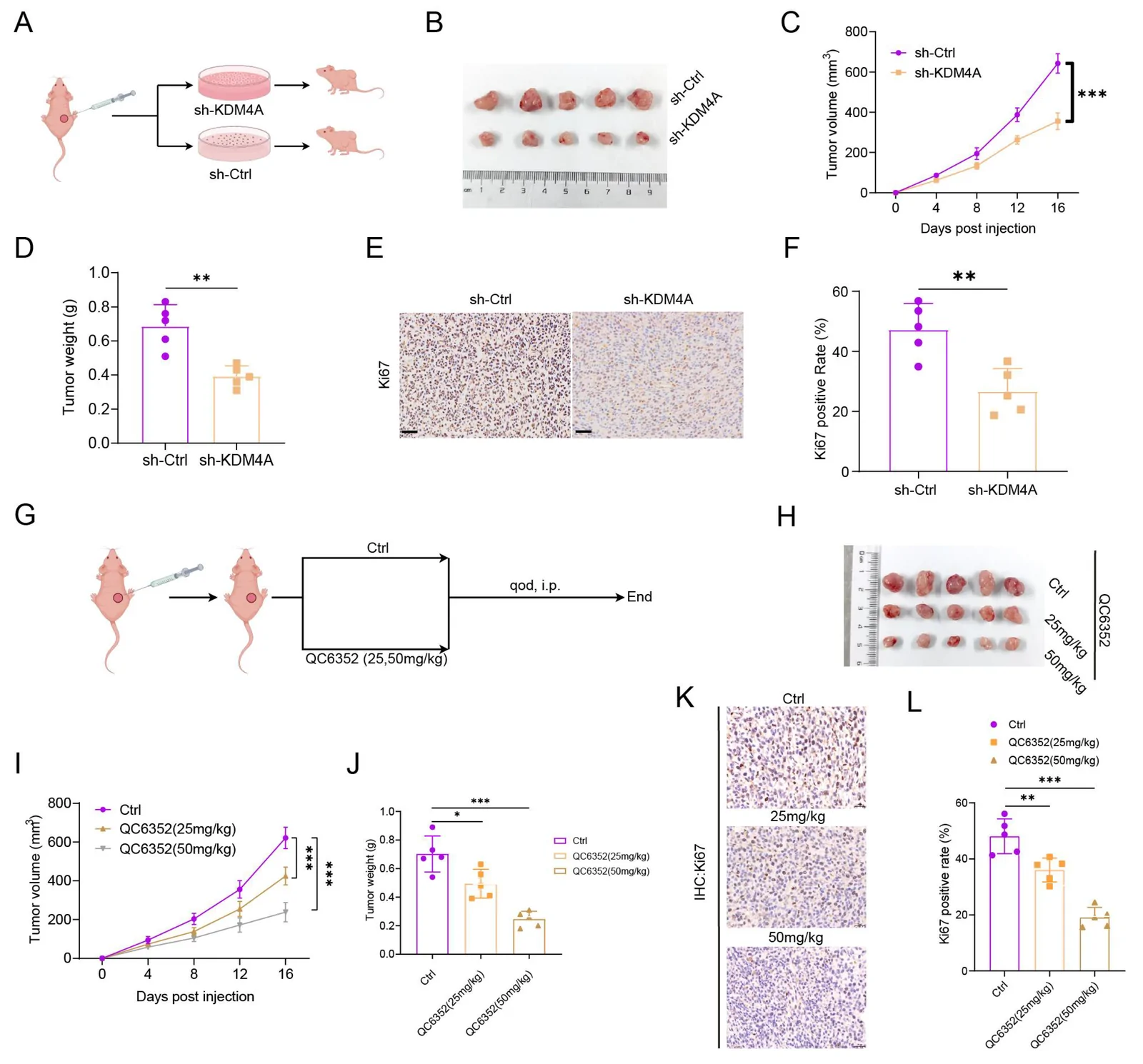
** KDM4A knockdown and QC6352 treatment suppress tumor growth *in vivo*. A.** Schematic diagram of the *in vivo* experimental design. HEC1-B cells stably expressing sh-Ctrl or sh-KDM4A were subcutaneously injected into BALB/c nude mice. **B.** Representative images of tumor tissues from the sh-Ctrl and sh-KDM4A group. **C.** Tumor volumes were monitored every 4 days after cell inoculation. **D.** Tumor weights were measured at the experimental endpoint. **E-F.** Representative images and quantification of IHC analysis of Ki67 protein expression in tumor tissues from BALB/c nude mice.** G.** Schematic diagram illustrating the treatment regimen of QC6352 (0, 25, 50 mg/kg) in a nude mouse model. **H.** Representative images of xenograft tumors from nude mice treated with increasing concentrations of QC6352.** I.** Tumor volumes were monitored every 4 days during QC6352 treatment. **J.** Tumor weights were measured at the end of treatment. **K-L.** Representative images and quantification of IHC analysis of Ki67 protein expression in tumors from nude mice treated with QC6352. n = 5 per group, Scale bar: 25 μm. *P < 0.05; **P < 0.01; ***P < 0.001.

**Figure 8:**
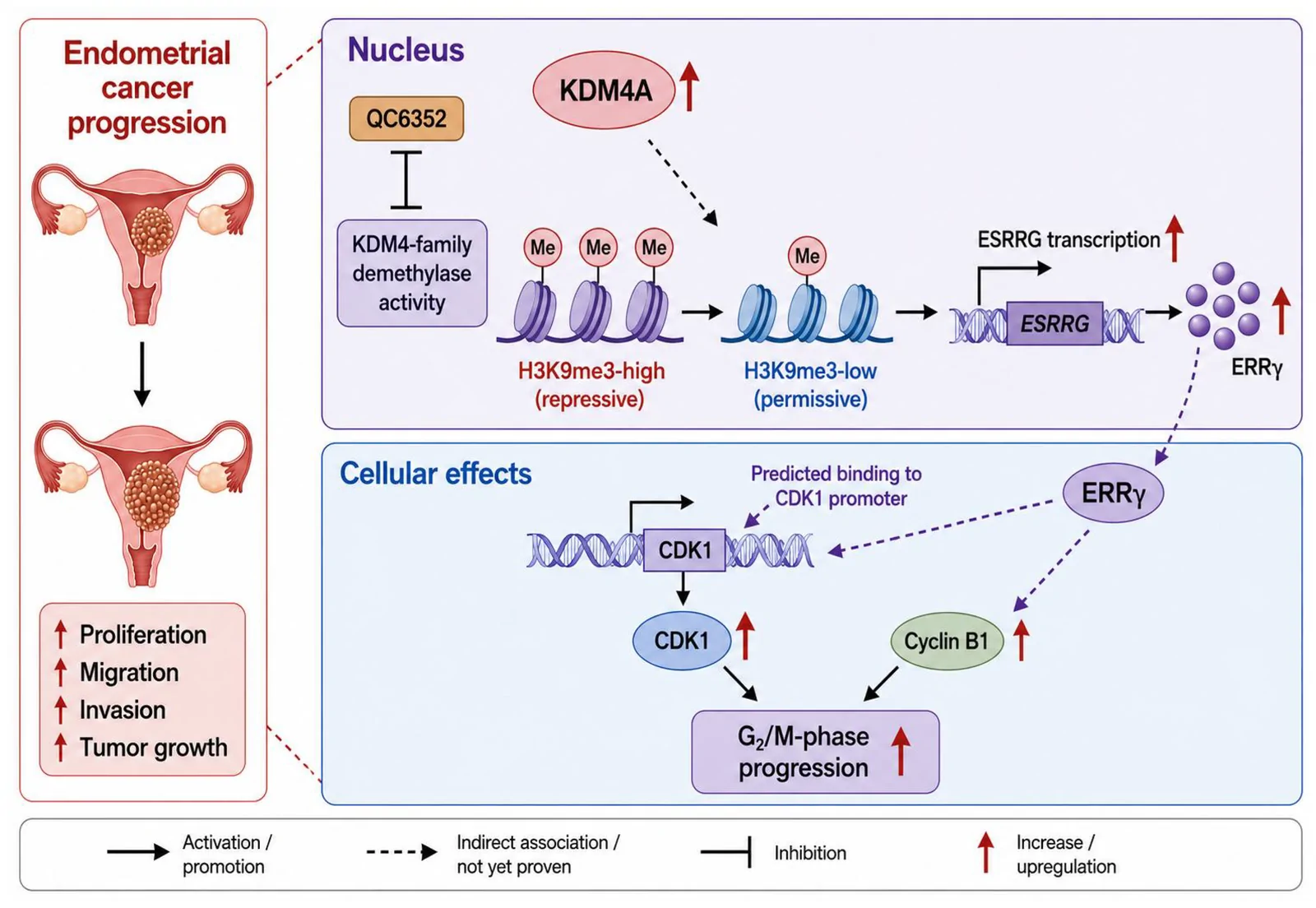
** Proposed model of the KDM4A-H3K9me3-ERRγ-associated cell-cycle regulatory axis in EC.** Elevated KDM4A expression is associated with reduced H3K9me3 enrichment at the ESRRG promoter and increased ERRγ expression. ERRγ is associated with increased CDK1 transcription and elevated Cyclin B1/CDK1 expression, which may contribute to altered G2/M-phase progression and enhanced malignant cellular phenotypes. QC6352 treatment suppresses EC cell growth and axis-related protein expression *in vitro* and reduces xenograft tumor growth *in vivo*.

## Data Availability

The ChIP-seq data generated in this study have been deposited in the Gene Expression Omnibus (GEO) database under accession number GSE319248. All necessary data can be obtained from the corresponding authors.

## Abbreviations

EC: Endometrial Cancer
KDM4A: Lysine Demethylase 4A
ERRγ: Estrogen-Related Receptor Gamma
TCGA: The Cancer Genome Atlas
GEO: Gene Expression Omnibus
ChIP-seq: Chromatin Immunoprecipitation Sequencing
IHC: Immunohistochemistry
ChIP-qPCR: Chromatin Immunoprecipitation Quantitative PCR
FDR: False Discovery Rate
GEPIA: Gene Expression Profiling Interactive Analysis
EMT: Epithelial-Mesenchymal Transition
qRT-PCR: Quantitative Reverse Transcription Polymerase Chain Reaction
CCK-8: Cell Counting Kit-8
ChIP: Chromatin Immunoprecipitation
mIHC: Multiple Immunohistochemistry
ROC: Receiver Operating Characteristic
AUC: Area Under Curve
