# Supplementary material for: Histone demethylase KDM4A promotes endometrial cancer progression through an ERRγ-associated cell-cycle regulatory axis
Source: Int J Biol Sci. 2026 Sep 3;22(14):7827–44. doi: 10.7150/ijbs.133986 (PMC13617999; doi:10.7150/ijbs.133986)
Supplement: Supplementary file 1 [file ijbsv22p7827s1.pdf]

# Histone demethylase KDM4A promotes endometrial cancer progression through an ERRγ-associated cell-cycle regulatory axis

Junfeng Chen[1#], Xiaoli Wen[1#], Donghai Zhang[1], Mengyue Zhu[1], Hong Zhou[1], Yiran Li[1,2**], Xiaoping Wan[1,3*]

[1] Shanghai Key Laboratory of Maternal Fetal Medicine, Shanghai Institute of Maternal-Fetal Medicine and Gynecologic Oncology, Shanghai First Maternity and Infant Hospital, School of Medicine, Tongji University, Shanghai, 200092, China

[2] Center for Reproductive Medicine, Shanghai First Maternity and Infant Hospital, Tongji University School of Medicine, Tongji University, Shanghai 200092, China. liyiran2007@gmail.com.

[3] Department of Gynecology, Shanghai First Maternity and Infant Hospital, School of Medicine, Tongji University, Shanghai, China. wanxiaoping@tongji.edu.cn.

[#]These authors contributed equally to this work.

*Corresponding author: Shanghai Key Laboratory of Maternal Fetal Medicine, Shanghai Institute of Maternal-Fetal Medicine and Gynecologic Oncology, Clinical and Translational Research Center, Shanghai First Maternity and Infant Hospital, School of Medicine, Tongji University, Shanghai, China. E-mail addresses: wanxiaoping@tongji.edu.cn.

**Corresponding author: Center for Reproductive Medicine, Shanghai

First Maternity and Infant Hospital, Tongji University School of Medicine, Tongji University, Shanghai 200092, China. E-mail addresses: liyiran2007@gmail.com.

## Supplementary tables

**Supplementary Table S1.** Antibodies used in this study

| Antibody | Catalog | Company |
|---|---|---|
| KDM4A | ab191433 | Abcam |
| ERRγ | sc-376449 | Santa Cruz |
| ERRγ | HY-P82264 | MedChemExpress |
| GAPDH | ab9485 | Abcam |
| Cyclin B1 | A16038 | ABclonal |
| CDK1 | A0220 | ABclonal |
| TriMethyl-Histone H3-K9 | A22295 | ABclonal |
| TriMethyl-Histone H3-K36 | A20379 | ABclonal |
| Histone H3 | A2348 | ABclonal |
| N-cadherin | ab76011 | Abcam |
| E-cadherin | A3044 | ABclonal |
| Vimentin | 5741 | CST |
| IgG antibody | ab172730 | Abcam |
| Ki67 | ab15580 | Abcam |
| HRP-conjugated Goat anti-Rabbit IgG (H+L) | AS014 | ABclonal |

**Supplementary Table S2.** Target sequences of shRNAs and primer sequences used for the construction of KDM4A and ESRRG overexpression vectors

**shRNA target sequences**

| Gene | Sequence |
|---|---|
| **sh-KDM4A#1** | GCACCGAGTTTGTCTTGAAAT |
| **sh-KDM4A#2** | TTCGAGAGTTCCGCAAGATAG |
| **sh-KDM4A#3** | GACTGCTGTTTATGCTCATTA |
| **sh-ESRRG#1** | CGGTCTCTTTCGTTTGAGGAT |
| **sh-ESRRG#2** | CCCTTATTTCTTTGCTTCTTT |
| **sh-ESRRG#3** | CGAATGAATGTGAAATCACAA |

**Cloning primers for overexpression constructs**

| Gene | | Sequence |
|---|---|---|
| **KDM4A** | **Forward:** | ATGGCCAGCGAGAGCGAGACA |
| | **Reverse:** | TTCCATGATGGCCCGATACAGAGCG |
| **ERRγ** | **Forward:** | CAGAACGGACTTGACTCGCC |
| | **Reverse:** | CCCAGAAGCGATGTCACCAC |

**Supplementary Table S3.** The sequences of gene-specific primers used for qRT-PCR, and ChIP assays

| **Gene name** | **Forward (5'-3')** | **Reverse (5'-3')** |
|---|---|---|
| **Primers for qRT-PCR** | | |
| GAPDH | GGCTGTTGTCATACTTCTCATGG | GGAGCGAGATCCCTCCAAAAT |
| CDK1 | GGAAATTGAGCGGAGAGCGA | TGGCTACCACTTGACCTGTAG |
| ESRRG | CTACCCTTCTGCTCCTATCCTG | AGCGATGTCACCACACACTAAA |
| | | |
| **Primers for ChIP** | | |
| *ESRRG* promoter | AGAGGGATGGTGCCAAATGG | AAGACTGGCCTTTACGTGGG |

**Supplementary Table S4.** Association between KDM4A expression groups and clinicopathological characteristics in UCEC cohorts

| Dataset | Clinical | Type | Total | High | Low | P-value |
|---|---|---|---|---|---|---|
| TCGA | Age | >65 | 224(42.83%) | 111(42.53%) | 113(43.13%) | >0.999 |
| | | <=65 | 297(56.79%) | 148(56.7%) | 149(56.87%) | |
| | | unknown | 2(0.38%) | 2(0.77%) | 0(0%) | |
| | Grade | G1 | 98(18.74%) | 64(24.52%) | 34(12.98%) | <0.001 |
| | | G2 | 116(22.18%) | 73(27.97%) | 43(16.41%) | |
| | | G3 | 298(56.98%) | 119(45.59%) | 179(68.32%) | |
| | | G4 | 11(2.1%) | 5(1.92%) | 6(2.29%) | |
| | Stage | I | 325(62.14%) | 189(72.41%) | 136(51.91%) | <0.001 |
| | | II | 48(9.18%) | 22(8.43%) | 26(9.92%) | |
| | | III | 121(23.14%) | 44(16.86%) | 77(29.39%) | |
| | | IV | 29(5.54%) | 6(2.3%) | 23(8.78%) | |
| GSE106191 | Age | >65 | 4(6.25%) | 0(0%) | 4(12.5%) | 0.121 |
| | | <=65 | 60(93.75%) | 32(100%) | 28(87.5%) | |
| GSE17025 | Age | >65 | 19(20.88%) | 6(13.33%) | 13(28.26%) | 0.031 |
| | | <=65 | 55(60.44%) | 35(77.78%) | 20(43.48%) | |

| Dataset | Clinical | Type | Total | High | Low | P-value |
|---|---|---|---|---|---|---|
| | | unknown | 17(18.68%) | 4(8.89%) | 13(28.26%) | |
| | Grade | G1 | 30(32.97%) | 9(19.57%) | 21(46.67%) | 0.003 |
| | | G2 | 36(39.56%) | 18(39.13%) | 18(40%) | |
| | | G3 | 25(27.47%) | 19(41.3%) | 6(13.33%) | |
| | Stage | IA | 30(32.97%) | 15(33.33%) | 15(32.61%) | 0.728 |
| | | IB | 37(40.66%) | 17(37.78%) | 20(43.48%) | |
| | | IC | 23(25.27%) | 13(28.89%) | 10(21.74%) | |
| | | unknown | 1(1.1%) | 0(0%) | 1(2.17%) | |

## Supplementary figures and figure legends

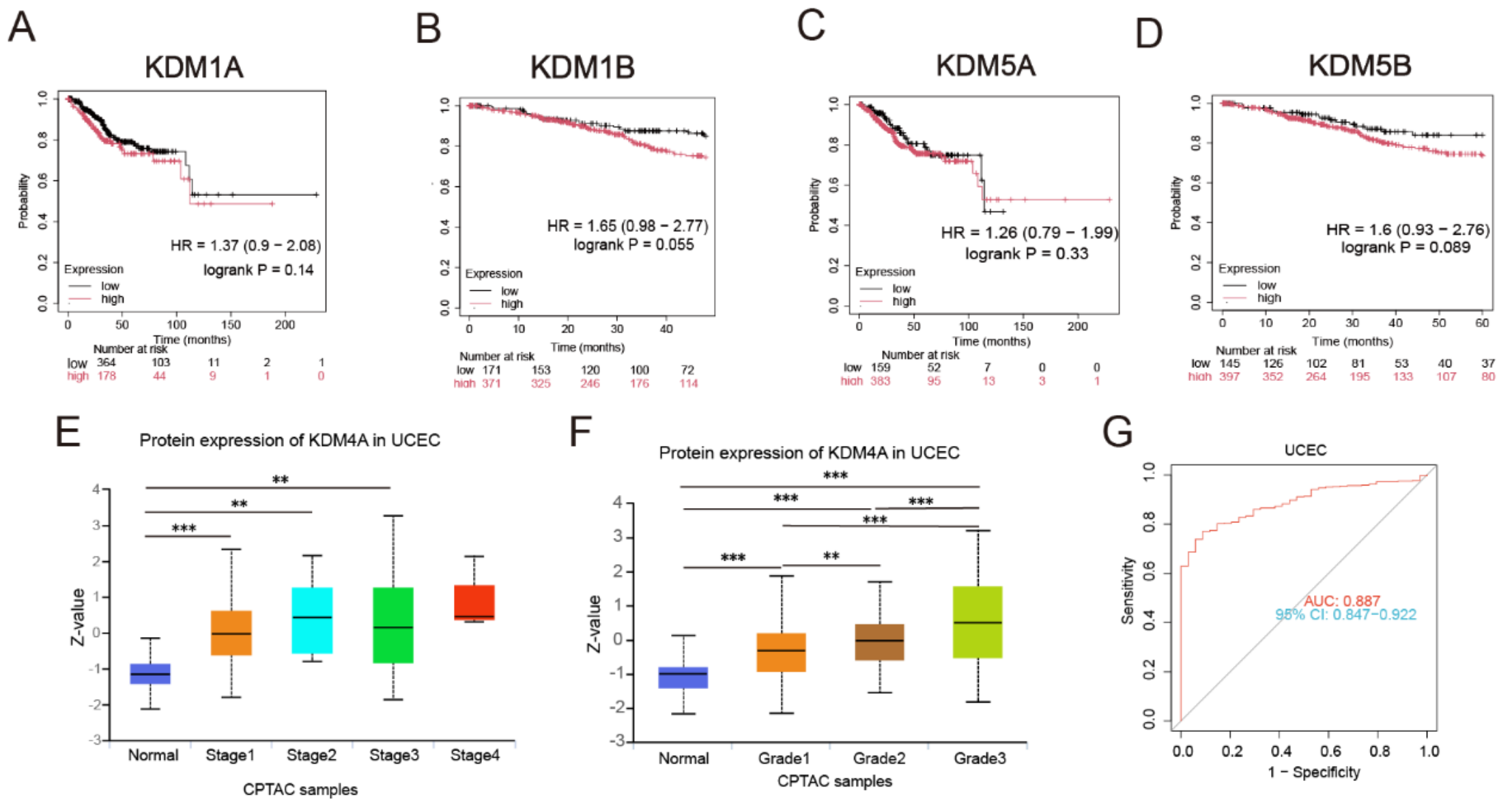


**Figure S1. Prognostic significance of KDM1A, KDM1B, KDM5A, and KDM5B and the clinicopathological relevance of KDM4A in UCEC.**

(A–D) Kaplan–Meier analyses of overall survival in patients with UCEC stratified into high- and low-expression groups according to the expression levels of KDM1A (A), KDM1B (B), KDM5A (C), and KDM5B (D). Hazard ratios (HRs) with 95% confidence intervals and log-rank *P* values are shown. (E) CPTAC-based comparison of KDM4A protein expression between normal endometrial tissues and EC tissues stratified by pathological stage (Stages I–IV). (F) Comparison of KDM4A protein expression between normal endometrial tissues and EC tissues stratified by histological grade (Grades 1–3). (G) Receiver operating characteristic (ROC) curve evaluating the ability of KDM4A expression to distinguish EC tissues from normal tissues. The area under the curve (AUC) was 0.887 (95% CI, 0.847–0.922). **P < 0.01 and ***P < 0.001.

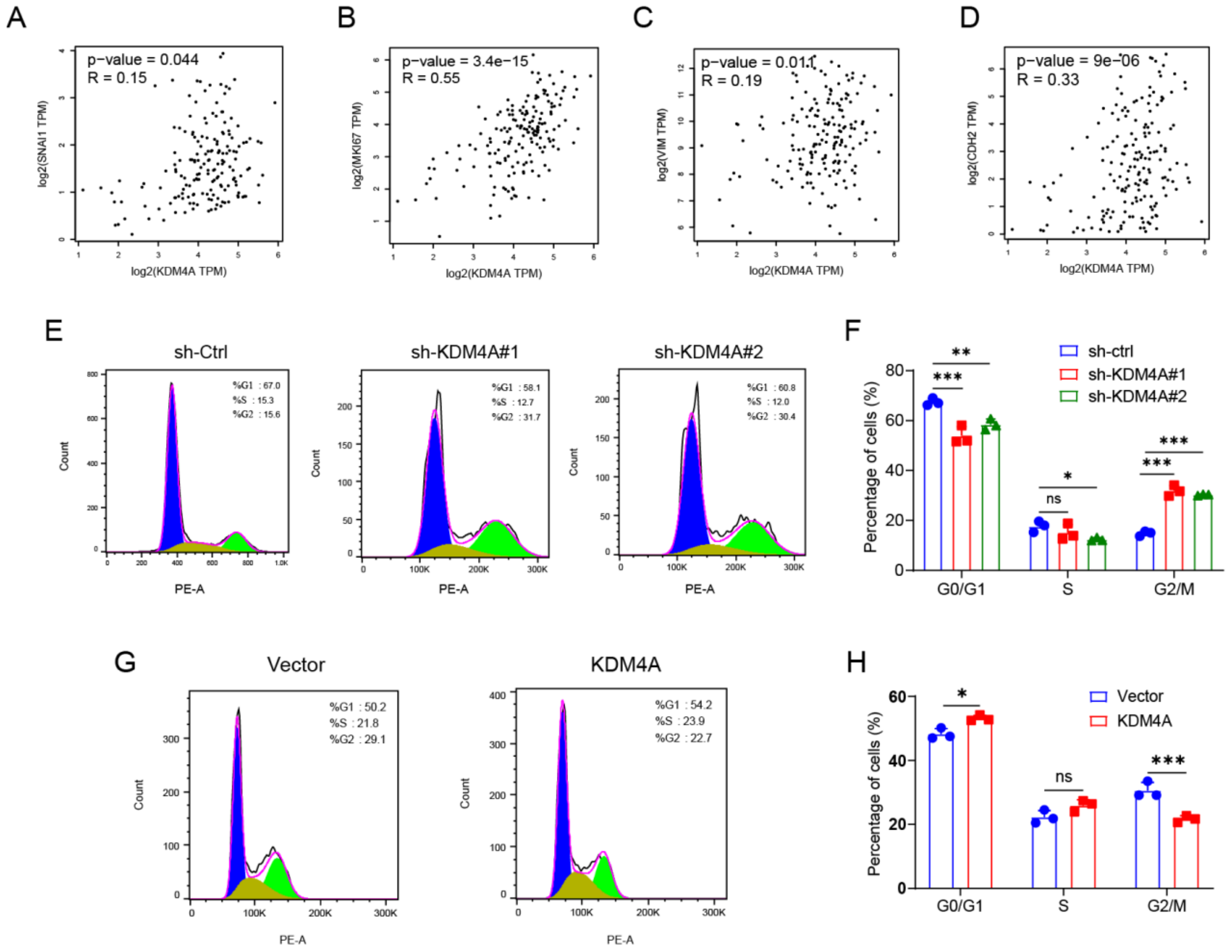


**Figure S2. KDM4A expression is associated with proliferation- and EMT-related markers and regulates cell-cycle distribution in endometrial cancer cells.**

(A–D) Correlation analyses of KDM4A expression with SNAI1 (A), MKI67 (B), VIM (C), and CDH2 (D) expression in UCEC samples. The correlation coefficient (R) and corresponding P value are indicated in each plot. (E, F) Representative flow-cytometric profiles (E) and quantitative analysis (F) of cell-cycle distribution in Ishikawa cells transduced with control shRNA (sh-Ctrl) or two independent KDM4A-targeting shRNAs (sh-KDM4A#1 and sh-KDM4A#2). (G, H) Representative flow-cytometric profiles (G) and quantitative analysis (H) of cell-cycle distribution in HEC1-B cells transduced with the empty vector or a KDM4A-overexpression construct. Data are presented as the mean ± SD. ns, not significant; * $P < 0.05$; ** $P < 0.01$; *** $P < 0.001$.

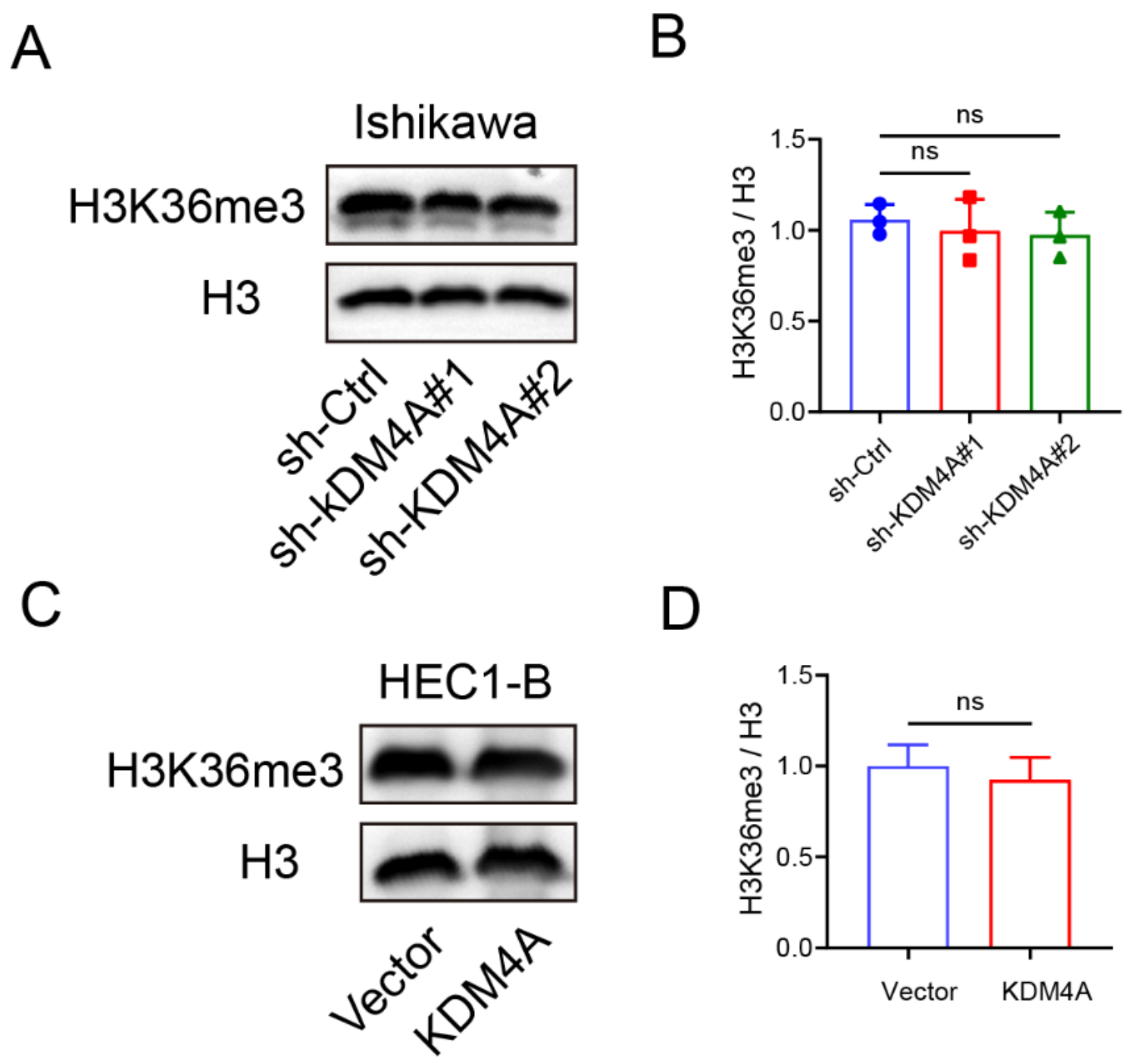


**Figure S3. The effect of KDM4A on H3K36me3 levels in EC cells.**

(A-B) Western blot analysis of H3K36me3 expression in Ishikawa cells transfected with KDM4A knockdown (sh-KDM4A#1, sh-KDM4A#2) or control (sh-Ctrl) constructs (A). Quantification of H3K36me3 relative to H3 expression in Ishikawa cells (B). (C-D) Western blot analysis of H3K36me3 expression in HEC1-B cells transfected with KDM4A overexpression or vector control constructs (C). Quantification of H3K36me3 relative to H3 expression in HEC1-B cells (D). Data are presented as the mean ± SD. ns, not significant.

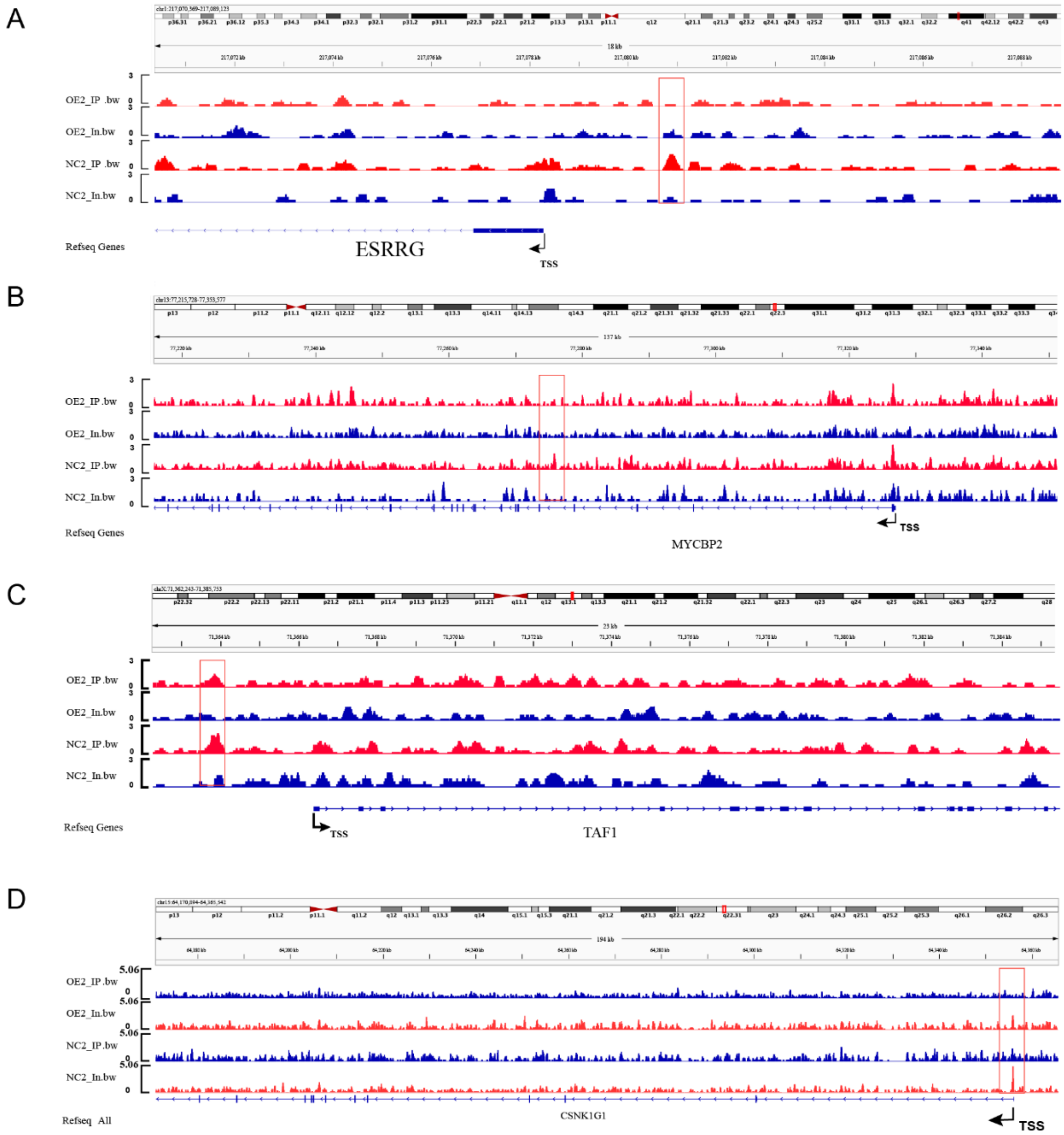


**Figure S4. Genome browser visualization of H3K9me3 ChIP-seq signals at selected genomic loci following KDM4A overexpression.**

(A–D) Representative IGV tracks showing H3K9me3 ChIP-seq enrichment and the corresponding input signals in KDM4A-overexpressing (OE2) and negative-control (NC2) cells at the ESRRG (A), MYCBP2 (B), TAF1 (C), and CSNK1G1 (D) loci. Red boxes indicate regions selected for visual comparison and subsequent ChIP-qPCR validation. Gene structures and transcription start sites (TSSs) are shown below the

signal tracks. IP, immunoprecipitated DNA; In, input DNA; bw, bigWig signal track.

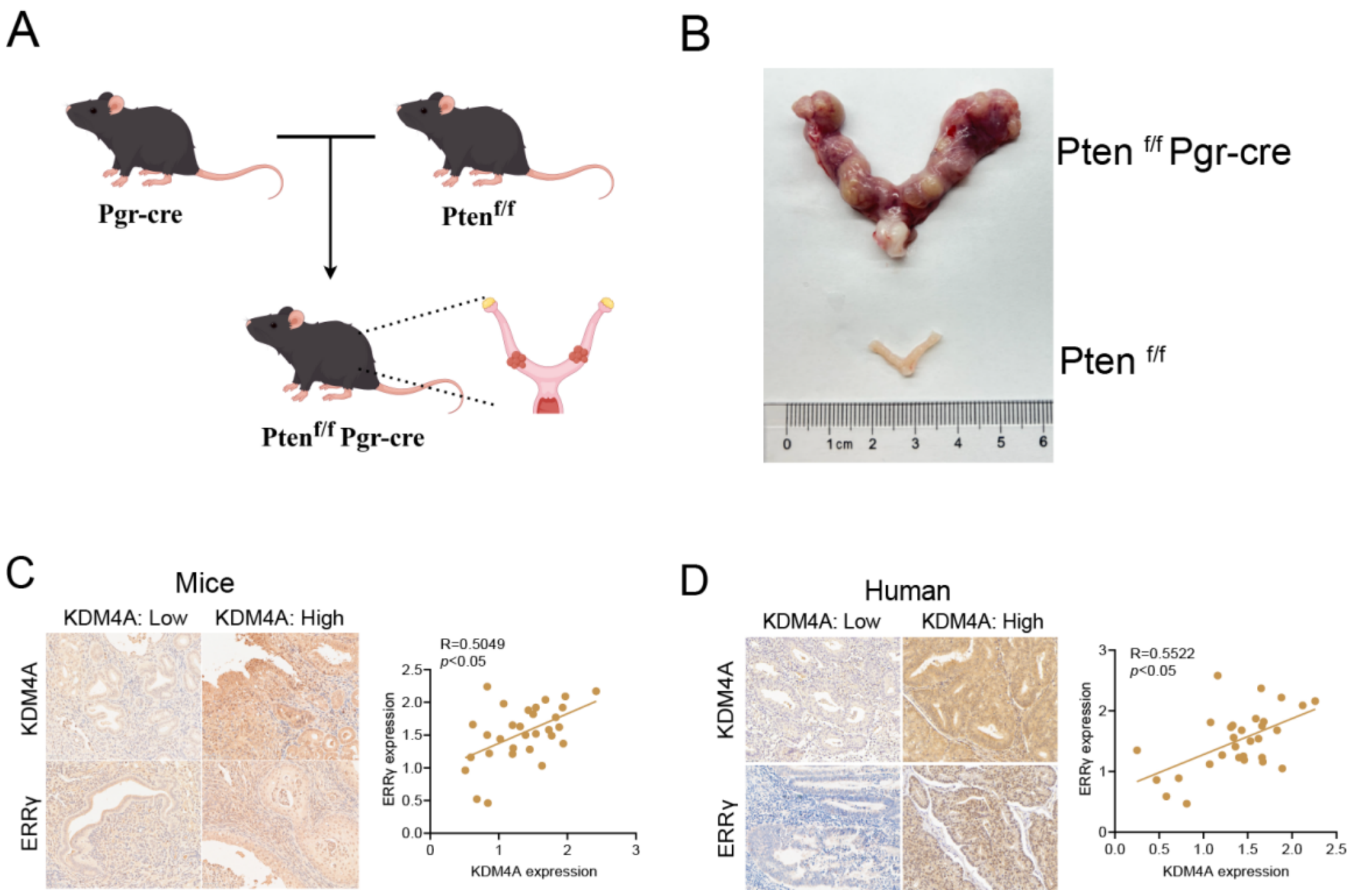


**Figure S5. KDM4A expression is positively associated with ERRγ expression in mouse and human EC tissues.**

(A) Schematic illustration of the generation of uterus-specific Pten-deficient mice by crossing Pgr-Cre mice with Pten$^{f/f}$ mice. (B) Representative gross images of uteri from mice with the indicated genotypes. (C) Representative immunohistochemical staining of KDM4A and ERRγ in mouse EC tissues with low or high KDM4A expression, together with correlation analysis between KDM4A and ERRγ expression levels. (D) Representative immunohistochemical staining of KDM4A and ERRγ in human EC tissues with low or high KDM4A expression, together with correlation analysis between KDM4A and ERRγ expression levels. The correlation coefficients and corresponding p-values are indicated in the plots.

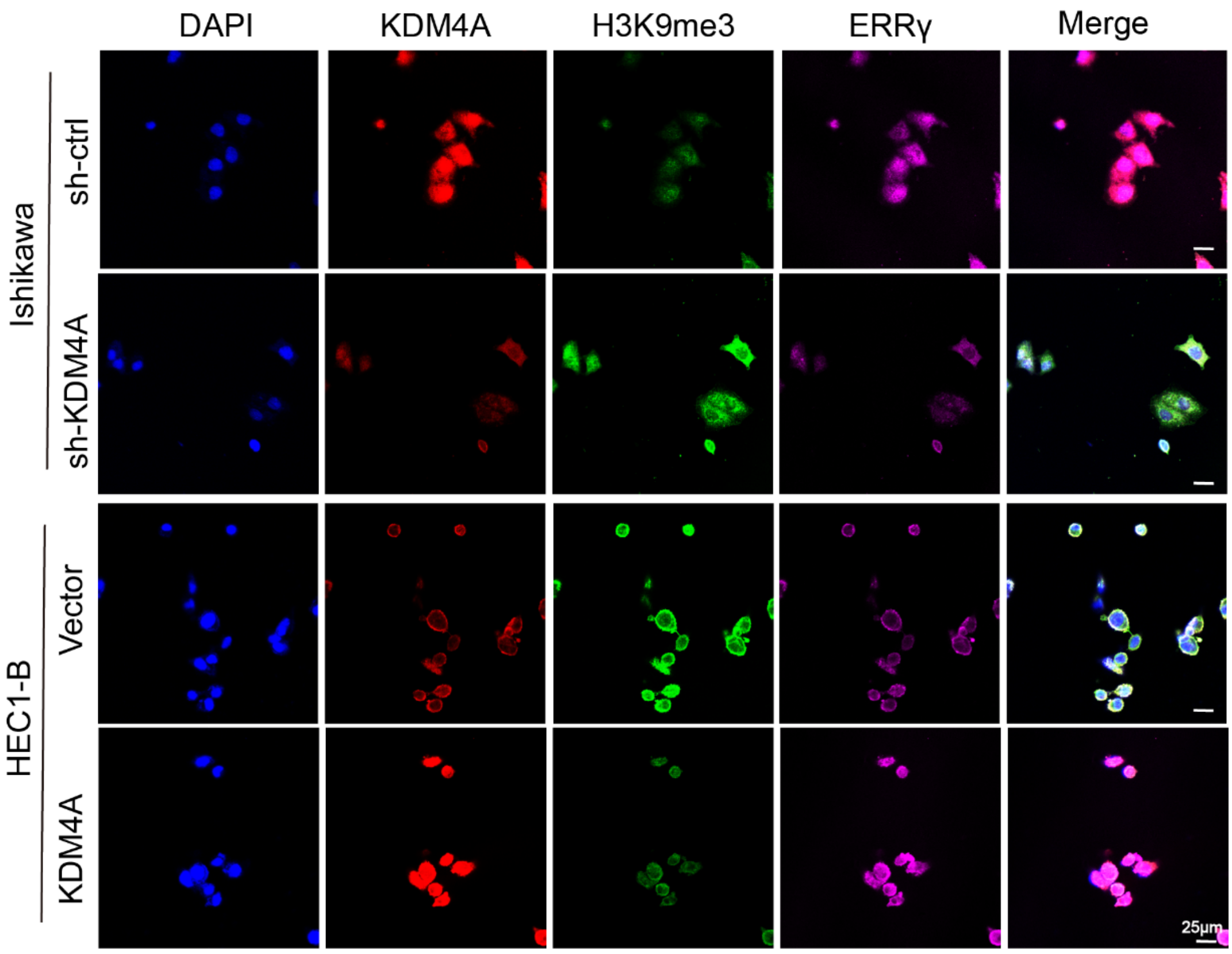


**Figure S6. Effects of KDM4A knockdown and overexpression on H3K9me3 and ERRγ immunofluorescence signals in EC cells.** Representative multiplex immunofluorescence images of KDM4A, H3K9me3, and ERRγ in Ishikawa cells transduced with sh-Ctrl or sh-KDM4A and in HEC1-B cells transduced with empty vector or a KDM4A-overexpression construct. KDM4A, H3K9me3, and ERRγ are shown in red, green, and magenta, respectively. Nuclei were counterstained with DAPI. Scale bar, 25 μm.

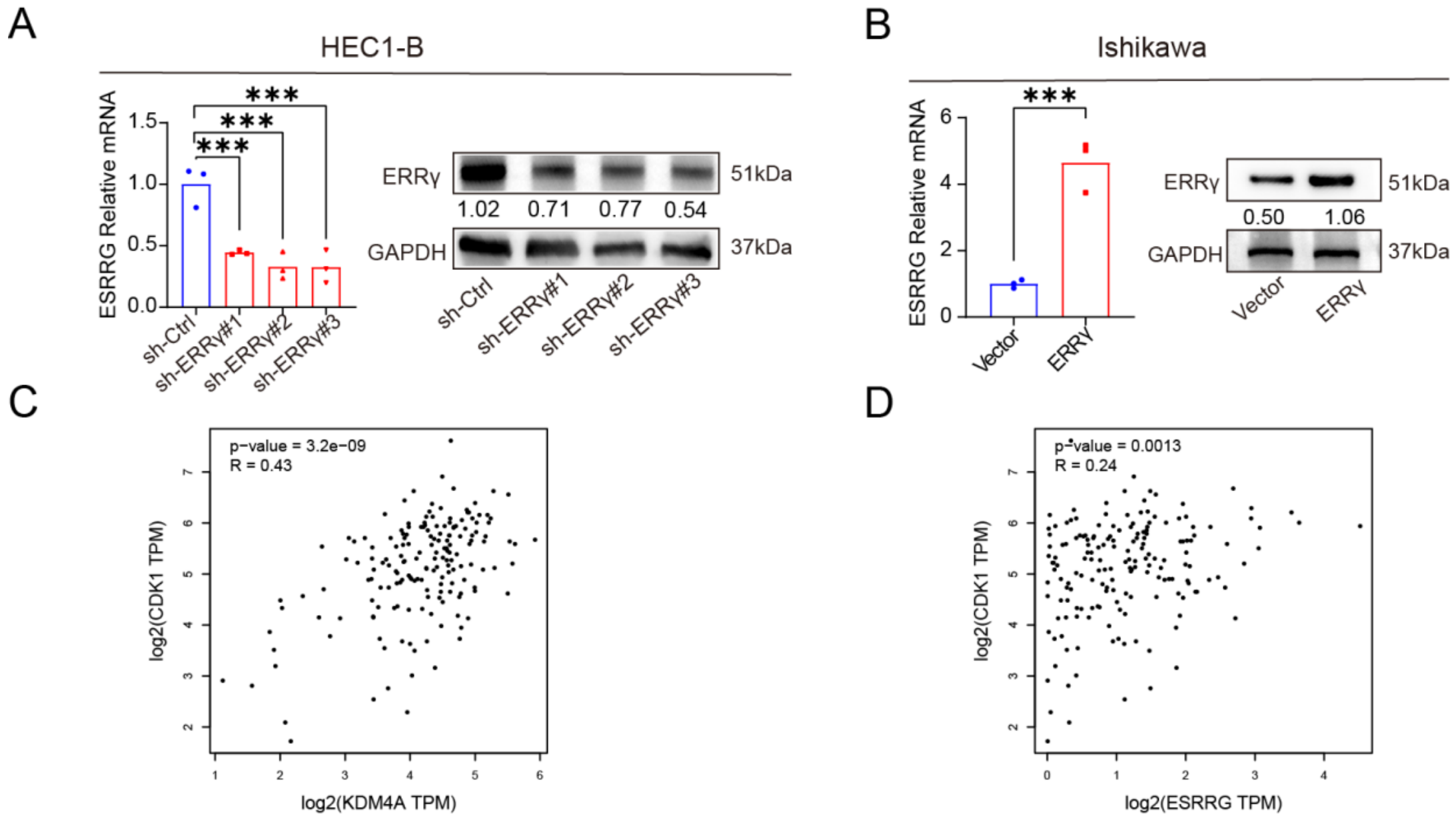


**Figure S7. Validation of ERRγ manipulation and expression correlations within the KDM4A–ERRγ–CDK1 axis.**

(A) Validation of ERRγ knockdown in HEC-1B cells transfected with sh-Ctrl or sh-ERRγ. ESRRG mRNA and ERRγ protein expression were assessed by quantitative real-time PCR and western blotting, respectively. (B) Validation of ERRγ overexpression in Ishikawa cells transfected with an empty vector or an ERRγ-overexpression plasmid. ESRRG mRNA and ERRγ protein levels were determined by quantitative real-time PCR and western blotting, respectively. (C) Correlation analysis between KDM4A and CDK1 mRNA expression in UCEC tissues. (D) Correlation analysis between ESRRG and CDK1 mRNA expression in UCEC tissues. Correlation coefficients (R) and corresponding p-values are indicated in the plots. Data are presented as the mean ± SD. ***P < 0.001.

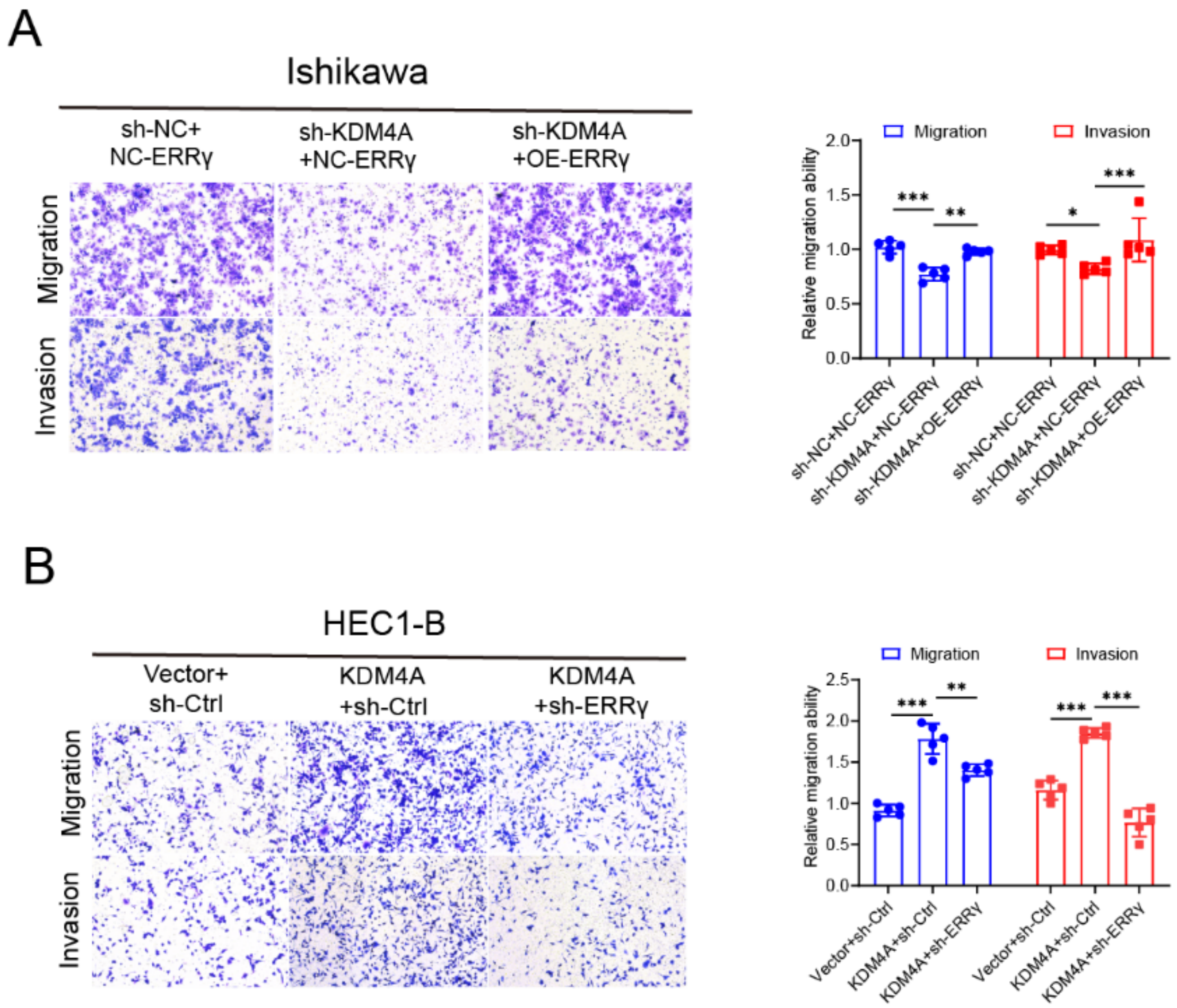


**Figure S8. ERRγ partially mediates KDM4A-associated migration and invasion in EC cells.**

(A) Representative images and quantitative analysis of Transwell migration and invasion assays in Ishikawa cells transduced with sh-NC + NC-ERRγ, sh-KDM4A + NC-ERRγ, or sh-KDM4A + OE-ERRγ. (B) Representative images and quantitative analysis of Transwell migration and invasion assays in HEC1-B cells transduced with Vector + sh-Ctrl, KDM4A + sh-Ctrl, or KDM4A + sh-ERRγ. Data are presented as the mean ± SD. * $P < 0.05$; ** $P < 0.01$; *** $P < 0.001$.
